# Stabilization of the retromer complex: Analysis of novel binding sites of bis-1,3-phenyl guanylhydrazone 2a to the VPS29/VPS35 interface

**DOI:** 10.1016/j.csbj.2024.02.026

**Published:** 2024-03-02

**Authors:** Elisa Fagnani, Francesco Bonì, Pierfausto Seneci, Davide Gornati, Luca Muzio, Eloise Mastrangelo, Mario Milani

**Affiliations:** a Biophysics Institute, CNR-IBF, Via Corti 12, I-20133 Milano, Italy; b Department of Bioscience, University of Milan, Via Celoria 26, I-20133 Milano, Italy; c Department of Chemistry, University of Milan, Via Celoria 26, I-20133 Milano, Italy; d INSPE—Institute of Experimental Neurology, San Raffaele Scientific Institute, Via Olgettina 60, I–20132 Milano, Italy

**Keywords:** Crystal structure, Molecular dynamics simulations, Protein ligand interaction, Protein binding sites

## Abstract

The stabilization of the retromer protein complex can be effective in the treatment of different neurological disorders. Following the identification of bis-1,3-phenyl guanylhydrazone **2a** as an effective new compound for the treatment of amyotrophic lateral sclerosis, in this work we analyze the possible binding sites of this molecule to the VPS35/VPS29 dimer of the retromer complex. Our results show that the affinity for different sites of the protein assembly depends on compound charge and therefore slight changes in the cell microenvironment could promote different binding states. Finally, we describe a novel binding site located in a deep cleft between VPS29 and VPS35 that should be further explored to select novel molecular chaperones for the stabilization of the retromer complex.

## 1 Introduction

Misfolded proteins and their aggregation are pathological hallmarks of different neurodegenerative disorders such as Parkinson’s disease (PD) and Alzheimer’s disease (AD) [20]. In Amyotrophic Lateral Sclerosis (ALS) several evidences indicate that the accumulation of misfolded and aggregated proteins in motor neurons (MNs) may affect the axonal transport [4] resulting in cell death. Strategies fostering the clearance of protein aggregates exert beneficial effects in ALS preclinical models, increasing MN survival.

The retromer complex (RC) controls the cellular localization and homeostasis of hundreds of transmembrane proteins [24,21,26], being involved in their recycling from early and maturing endosomes either to the trans-Golgi network (TGN) or back to the plasma membrane [22]. The RC depends on transient association of the cargo-selective heterotrimer, formed by vacuolar protein sorting (VPS) 35, VPS26 and VPS29, with a few sorting nexins (Snx) components (Snx1 or 2 assembled with Snx5 or 6 forming a heterodimer [23]). Being a key element in endosomal trafficking, retromer malfunction is related to major neurodegenerative disorders [28,2].

The identification of small molecules able to enhance the stability of the RC, and particularly of the cargo selective trimer, is considered an important therapeutic option [13,10]. VPS35 (92 kDa) is the central protein component of the cargo selective trimer and is characterized by an elongated solenoid-like alpha helical structure that acts as a scaffold for the binding of VPS26 [25] - at its N-terminal portion - and VPS29 at the C-terminal end [7]. Since VPS26 and VPS29 do not interact with each other, the strategies to identify molecular chaperones to stabilize the complex are focused on the stabilization of either the VPS35/VPS29 or VPS35/VPS26 interfaces.

In our previous work, starting from the isothiourea R55 - the first molecular chaperone for retromer stabilization [13] - we identified a new class of compounds acting as stabilizers of the VPS35/VPS29 interface [15]. Despite the promising activity of the early lead bis-1,3-phenyl guanylhydrazone (hereafter **2a**), an important information that is still missing is the experimental identification of **2a** binding site(s) on VPS35/VPS29.

In this work we present the results of two X-ray diffraction experiments on crystals of the VPS35/VPS29 complex in presence of **2a**. The electron density maps allowed the identification of two novel potential binding sites (named *site1* and *site2*) beyond the known R55 binding site (*site3*, located approximately between VPS35 Gln538 and VPS29 Ile146 [13]). The interaction of **2a** with these three possible binding sites has been further analyzed with molecular dynamics (MD) simulations. Our computational analysis showed a different behavior of the ligand when modeled as neutral (**2a**), single charged (**2a**^**+**^) or fully charged (**2a**^**+2**^). Taken together, our results show *site1* as a novel promising binding site for molecular chaperones to stabilize the VPS35/29 interface, to be further investigated for the selection of novel active molecules.

## 2 Methods

### 2.1 VPS35 deletion mutagenesis

Plasmids for the expression of VPS29 (pMR101-VPS29) [7,11] and VPS35 (pGST-parallel2) were kindly provided by Dr. Aitor Hierro. The cDNA coding for human VPS35 domain was cloned in pGST-parallel2 vector-based construct as a fusion with a cleavable N-terminal Glutathione S-transferase tag. A two-step mutagenesis experiment was performed to delete 474 residues at the N-terminal of the protein (VPS35C, M475-Leu796). Thereafter, we deleted 16 additional amino acids at the C-terminal extremity of VPS35 (VPS35sh from M475 to Arg780), using the Q5 Site-directed mutagenesis kit (New England Biolabs), in which forward and reverse primers work separately, each amplifying a different DNA strand. The primers designed for the mutations were:

VPS35-C fwd 5′- GTAGAAGACCCTGATCCAG-3′.

rev 5′- CATGGCGCCCTGAAAATA-3′.

VPS35sh fwd 5′-GCGCTTGCGGTAAGAATCACCAG-3′.

rev 5′-AAATGCTCCAGTGTGTTATG-3′.

### 2.2 Expression and purification of VPS29/35

*Escherichia coli* BL21 (DE3) pLys (Invitrogen) competent cells were co-transformed with plasmids encoding for VPS29 (pMR101-VPS29) and VPS35C or VPS35sh. Bacterial cultures were grown at 37 °C in Luria-Bertani (LB) medium supplemented with ampicillin (100 μg/ml), kanamycin (30μg/ml) and chloramphenicol (34 μg/ml). Protein expression was induced at 20 °C by the addition of 0.5 mM IPTG, when cellular OD_600_ reached 1. After 16 h cells were harvested, resuspended in lysis buffer containing 50 mM Tris-HCl pH 8.0, 300 mM NaCl, 1 mM DTT, protease inhibitors, 20 µg/ml DNase, 40 mM MgSO_4_ and 100 µg/ml lysozyme, and disrupted firstly by sonication (to reduce the high viscosity of the sample) and then by French press treatment. The resulting extract was clarified by centrifugation at 39,000 RCF and loaded on a Glutathione Sepharose^TM^ 4B resin for affinity chromatography. The complex was eluted after the cleavage of GST with TEV protease in 50 mM Tris-HCl pH 8.0, 300 mM NaCl and 10 mM DTT buffer (Fig. S1). A further step of purification was performed by size exclusion chromatography (Superdex 200, GE Healthcare) in a buffer containing 50 mM Tris-HCl pH 8.0, 300 mM NaCl, 1 mM DTT (Fig. S1). The complex was concentrated for crystallization trials to 19 mg/ml (345 µM) with Amicon Ultra centrifugal filter (10 kDa cut-off) and stored at -80 °C.

### 2.3 Crystallization and data collection

Co-crystallization trials of VPS35C or sh/VPS29 (345 µM) in complex with compound **2a** (5 mM, synthesized as described in Muzio et al. [15]) were carried out with an Oryx4 nanodispenser robot (Douglas Instrument) using the sitting drop vapor-diffusion setup at 20 °C.

The VPS35C/VPS29 complex generated only crystals diffracting at low resolution (∼ 7 Å), likely due to the presence of a disordered C-terminal tail. On the contrary, two well diffracting crystals (i.e. *A* and *B*) were obtained with the VPS35sh/VPS29 complex. The *A* crystal grew in 20% PEG 3350, 150 mM NaK tartrate, 100 mM NaCl, pH 7.4, whereas the *B* crystal in 18% PEG 3350, 150 mM NaK tartrate, pH 7.4. The crystals were cryo-protected with 25% glycerol and frozen in liquid nitrogen before data collection. Diffraction data were collected at beamline I04 at Diamond Light Source (Harwell Science and Innovation Campus in Oxfordshire; crystal *A*) and ID23eh2 at European Synchrotron Radiation Facility (Grenoble; crystal *B*).

### 2.4 Structure solution and analysis

The two datasets of VPS35sh/VPS29 with compound **2a** were indexed and scaled using *XDS* [8]. The structures were solved by molecular replacement (*MOLREP*, [27]) using the crystal structure of the VPS29/35 heterodimer (PDB: 2R17, [7]) as model. The refinement of the structures was performed with *REFMAC5* [14] and *BUSTER* (https://www.globalphasing.com) [3] and manually corrected using *COOT* [6]. The figures were prepared with *PyMol* (The PyMOL Molecular Graphics System, Version 2.5.0 Schrödinger, LLC).

### 2.5 Molecular dynamics simulations

Firstly, **2a** was analyzed using the "Marvin" package (version 22.4, ChemAxon (https://www.chemaxon.com)): the molecule was drawn with “Marvin Sketch”, all the tautomers were generated using "cxcalc", and the relative abundance of each tautomer at pH 7.4 was calculated with “Marvin Sketch” *pka* applet.

MD simulations were performed with the program GROMACS [17]. We started from the coordinates of the deposited PDB structure 2R17 (subunits A and C; [7]) using the GROMOS96 54a7 force field [19]; for each simulation, three molecules of double charged **2a**^**2+**^**,** charged **2a**^**+**^ or uncharged **2a** were manually placed nearby the *sites1–3* using the program COOT [6].

The uncharged / single charged / double charged ligands were parameterized with *Automated Topology Builder ATB3.0* (https://atb.uq.edu.au/; molid=1096183 / 1612934 / 307300) [12].

For **2a**^**2+**^ (system 1), the parallelepipedal box was filled with 17,699 water molecules and the system charge (-1) equilibrated with 21 and 20 atoms of Na^+^ and Cl^-^, respectively. After minimization with the steepest descent algorithm (final Potential Energy = -9.7529800e+05 kJ mol^−1^), we performed three serial equilibrations each lasting for 2 ns: 1. T = 100 K in NVT ensemble; 2. T = 300 K in NVT; and 3. T = 300 K, NPT ensemble, at atmospheric pressure.

For **2a** (system 2), the parallelepipedal box was filled with 16,430 water molecules and the system charge -7) equilibrated with 20 and 27 atoms of Na^+^ and Cl^-^, respectively. After minimization with steepest descent algorithm (final Potential Energy=-9.7878788e+05 kJ mol^−1^), we performed three serial equilibrations as already described for system 1.

Each of the nine productive runs performed either with the double charged or the uncharged ligands started from the same spatial configuration (one for system 1 and another for system 2) but with different velocity distributions (generated at T = 300 K), with Particle Mesh Ewald for long-range electrostatics, 2 fs time step (leap-frog integrator), T-coupling with modified Berendsen thermostat and P coupling Parrinello-Rahman, with periodic boundary conditions.

For the single charged ligand **2a**^**+**^ (system 3), the parallelepipedal box was filled with 17,733 water molecules and the system charge (-4) equilibrated with 20 and 24 atoms of Na^+^ and Cl^-^, respectively. After minimization with steepest descent algorithm (final Potential Energy=-9.7631775e+05 kJ mol^−1^), we performed two equilibrations in NVT ensemble each for 2 ns at T = 100 K and T = 300 K. In this case instead of nine independent runs, we performed a single run for 1 µs, with the same parameters described for system 1 and 2.

Each simulation was analyzed with the GROMACS package *cluster* [1], to cluster the conformations of the **2a** ligand in each site by using the single linkage method where a structure is added to the cluster when its distance to any element of the cluster is less than the chosen cutoff.

## 3 Results

### 3.1 Binding sites for 2a

VPS35sh/VPS29 was co-crystallized with compound **2a** [15] in different conditions (see Methods). The VPS35sh construct was chosen since the complex obtained with the longer C-terminal portion of the VPS35 protein (M475-Leu796) produced crystals that diffracted only at low resolution (∼7 Å, not shown).

In the asymmetric unit (a.u.) of the two crystals analyzed (*A* and *B*, both with orthorhombic symmetry; Table 1) two heterodimers of VPS35sh/VPS29 were present, albeit with differences in crystal packing as evidenced by the different values of unit cell parameters.

**Table 1:** X-ray data-collection and refinement statistics.

*Crystal*	*A*	*B*
Beam line & wavelength (Å)	DIAMOND i04-1 0.91788	ESRF id23eh2 0.87313
Space group	P2_1_2_1_2_1_	P2_1_2_1_2_1_
Unit-cell parameters (Å)	a= 57.5; b= 140.8; c= 141.3	a= 48.2; b= 130.3; c= 146.9
Heterodimers in a.u.	2	2
Resolution (Å)	19.9 – 2.5 (2.55-2.50)^a^	48.7 – 2.4 (2.45-2.40)^b^
Unique reflections	40,494 (2913)^a^	37,129 (2689)^b^
Completeness (%)	99.7 (99.9)	99.9 (100)
Redundancy	13.6 (14.3)	12.8 (13.3)
Rmeas^†^ (%)	9.2 (246.8)	39.8 (213.5)
CC(1/2) (%)	99.9 (59.7)	99.2 (50.6)
Average *I*/σ (*I*)	16.1 (1.1)	6.5 (1.1)
*Final model*		
R factor^‡^/Rfree^§^ (%)	20.2/26.4	18.8/24.8
r.m.s. bonds (Å)	0.008	0.006
r.m.s. angles (°)	1.44	1.42
Ramachandran favored / additionally allowed regions (%)	96/4%	97/3%
B-factors (VPS35)/(VPS29) [Å^2^]	(121.7, 112.3)/(114.1127.3)	(54.0, 61.4)/(54.8,52.2)
B-factor **2a** [Å^2^]	143.7	107.5
PDB-ID	8R02	8R0J
binding sites	*Site1*	*Site2*
Rohofit score & correl	-156 39%	-608 75%

Values in parentheses are for the highest resolution shell: ^a^(2.55-2.50), ^b^(2.45-2.40).

† R_meas_ = (Σ (n/(n-1) Σ |*I* - (*I*)|)/ Σ *I* x 100, where *I* is intensity of a reflection and (*I*) is its average intensity.

‡ R_factor_ = Σ |F_o_ - F_c_| / Σ |F_o_| x 100.

§ R_free_ is calculated on 5% randomly selected reflections, for cross-validation.

For both crystals, the Fo-Fc difference Fourier maps showed traces of additional electron density, where it was possible to model two **2a** molecules.

**Fig. 1:**
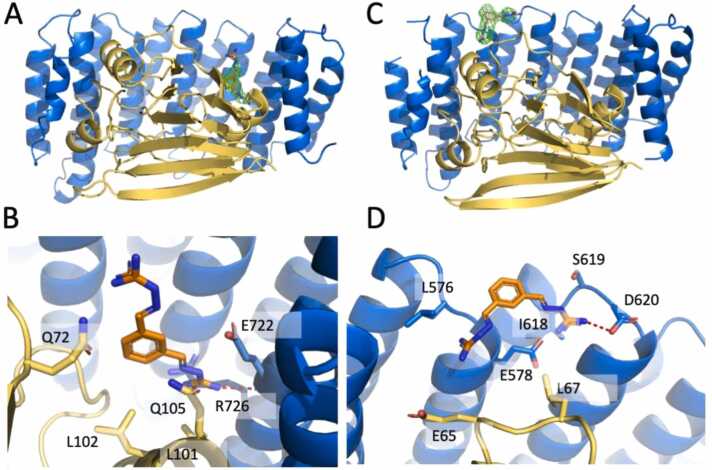
Crystal structures of VPS35sh/VPS29 (yellow/blue cartoons) in complex with **2a** (sticks with orange carbon atoms); (A, C) **2a** in *site1* and *2,* respectively, surrounded by 2Fo-Fc electron density contoured at 1 sigma (in green); (B, D) close view of *site1* and *2,* respectively, with selected amino acids evidenced (sticks with yellow/blue carbon atoms for VPS29/VPS35sh). H-bond/salt bridge interactions between **2a** and the main chain carbonyl of Glu722 (2.6 Å) in *site1* or Asp620 (2.7 Å) in *site2* are indicated with red dotted lines.

In the *A* crystal, the analysis of the residual electron density performed with the program *rhofit* (https://www.globalphasing.com/), showed a possible binding site for **2a** only in one of the two heterodimers, in a deep cleft between VPS29 (α-helix 3, amino acids (aa.) 96–106) and VPS35 (alpha helices α9 (aa. 663–678) and α11 (aa. 713–731); *rhofit* statistics: score -156 correlation: 39%; Fig. 1A). In this site (*site1)* the electron density of the ligand was more defined for its phenyl moiety and for the guanylhydrazone arm pointing toward the protein core (Fig. 1B), while the other arm displayed a higher conformational freedom. The more stable portion of **2a** is in close contact with residues Gln72, Leu101-Leu102 and Gln105 from VPS29, and Glu722 and Arg726 from VPS35. More in details, the phenyl moiety of **2a** displayed an amino aromatic interaction with Gln105 (4.3 Å; [5] [29]) and Gln72 (3.3 Å) whose amino moiety was at ∼3.4 Å distance from side arm nitrogen; further stabilization of the ligand arm was provided by the interaction with the side chain of Arg726 (3.1 Å) and the main chain carbonyl of Glu722 (2.6 Å) (Fig. 1, A and B).

The *B* crystal (Table 1) showed significant differences with respect to the published structure (PDB: 2R17 [7]), specifically in the conformation of the residue Thr144 of VPS29. Thr144 is located in a loop of sizable length (amino acids 132–149) close to the R55 binding site (here named *site3*), and the different conformation of the threonine is present only in one of the two copies of VPS29 in the crystal a.u. Such different conformation might be caused either by crystal contacts (being Thr144 at ∼3.4 Å from Pro188 from a symmetry related molecule), or by the presence of **2a** in *site3,* albeit with low occupancy. *Rhofit* analysis (https://www.globalphasing.com/) showed the possible presence of **2a** also in *site2*, located in a crevice of VPS35 between the Ala574-Ala577 loop (connecting VPS35 α4 to α5) and the Ile618-Asp620 loop (linking α6 to α7) (*rhofit* score −674.1, correlation: 63%) (Fig. 1, C and D).

### 3.2 Molecular dynamics simulations of VPS35sh/VPS29 in the presence of three molecules of 2a

The experimental electron density in our crystals suggested the presence of two possible binding sites for **2a** (*site1* and *site2*, Fig. 1) albeit in just one of the heterodimers in the a.u. and with high B-factors (Table 1) or low occupancy. We decided to further analyze the interaction of **2a** with these two binding sites together with the previously identified R55 binding site (*site3*) by molecular dynamics (MD) simulations.

**2a** can have different charge states at physiological pH. For instance, analysis with the *Marvin* package (*cxcalc*, Marvin version 22.4, ChemAxon (https://www.chemaxon.com)) showed that **2a** (with delocalized double bonds in the guanyl hydrazones) should be 50% with charge + 1, 28% not charged and 22% with charge + 2, at pH 7.4 (or 40% charge +1, 8% not charged, and 52% with charge +2 starting from **2a** with localized double bonds). Since the charge state of a ligand can change during the interaction with a protein [16] we initially chose to analyse **2a** in the two extreme cases: i.e. 0 and +2 charge.

We started from the coordinates of VPS35sh/VPS29 plus three **2a** ligands, parameterized either as double charged **2a**^**+2**^ (system 1) or uncharged **2a** (system 2), located close to each of the three identified binding sites. After minimization and equilibration of the two systems (see Methods), nine MD simulations (each lasting for 100 ns, for a total simulation time of 0.9 µs) were run for each system, starting from the same spatial coordinates (one for system 1 and another for system 2) but with different distributions of initial velocities.

#### 3.2.1 Double charged 2a^+2^

The analysis of the nine simulations was initially performed to identify the amino acids located at the minimal distance from each of the three **2a**^**+2**^ ligands for every time step (Fig. S2-4). The persistence of certain amino acids at the minimum distance from the ligand implies the presence of (quasi)stable conformations during the simulations. Among the three sites, the longer persistence - i.e. the higher binding stability of **2a**^**+2**^ - was observed for *site2* (Fig. S3). For all the nine simulations a persistent minimal distance was maintained with negatively charged residues Glu575, Glu578, Glu617, Glu620 of VPS35 and, rarely, with Glu65 of VPS29. On the contrary, **2a**^**+2**^ displayed the lower persistence in *site3* (i.e. lower apparent affinity) from where it often completely lost contact with the protein during different simulations (Fig. S4).

In general, the charged compound **2a**^**+2**^ appeared to be quite promiscuous [18], being able to interact with different parts of the complex, and, as expected, with negatively charged amino acids. Despite its binding stability, in *site2* **2a**^**+2**^ interacted almost solely with VPS35 residues and only transiently and for negligible amounts of time with Glu65 in VPS29. Such evidence suggested that **2a**^**+2**^ binding to *site2* is unable to strenghten the interaction between the two proteins and could likely have only a minor effect in the stabilization of the VPS29-VPS35 complex.

#### 3.2.2 Uncharged 2a

Contrary to what was observed for the charged ligand, the permanence of uncharged **2a** in a stable conformation in *site2* was negligible (Fig. S6), and the simulations showed *site1* and *site3* as the more stable binding sites (Fig. S5-7).

To further investigate this result for each simulation, all the conformations of **2a** in each binding site were grouped in clusters with cutoff of 0.4 Å (program *cluster;* GROMACS package [1]; see Methods). The reference structure of the most populated cluster was chosen as representative of the entire simulation for the selected binding site, and analyzed to identify all the amino acids in contact with the ligand (with program *LigPlus* [9]; asterisks in Table S1). With this analysis, it was possible to group together all the nine simulations and to associate to each amino acid the relative occurrence of the contact with **2a** in *site1* (Table S1) and *site3* (Table S2). The relative occurrence of the contact, was defined as the sum of the number of structures in each reference cluster, where the contact was present (asterisks in Table S1 and S2), divided by the total number of structures in all the nine clusters (total number of clusters in Table S1 and S2).

In *site1,* the phenyl moiety of the ligand was shifted by about 4 Å respect to its position in the crystal structure. As reported in Table S1, in *site1* **2a** interacted mainly with VPS35 residues Lys622 (relative occurrence 73.2%) and Thr629 (88.3%) of ɑ7, Arg668 (100%) of ɑ9, Glu722 (87.4%) and Arg726 (81.4%) of ɑ11 and His88-Gln89 of VPS29 (Table S1; Fig. S8, A and B).

In *site3,* already described as the binding site of R55 [13], **2a** is mostly in close contact with VPS35 residues located at the N-terminal end of the construct (ɑ1 - loop(497−503) - ɑ2 and ɑ3 (538−542)), and in particular with Arg499, Ser500, Pro503, Gln506, Arg542 (Fig. S8, C and D). Moreover, **2a** established contacts with an extended loop of VPS29 (loop(137−146)) and especially with Thr144 and Tyr139 (Table S2; Fig. S8, C and D).

#### 3.2.3 Single charged 2a^+^

**Fig. 2:**
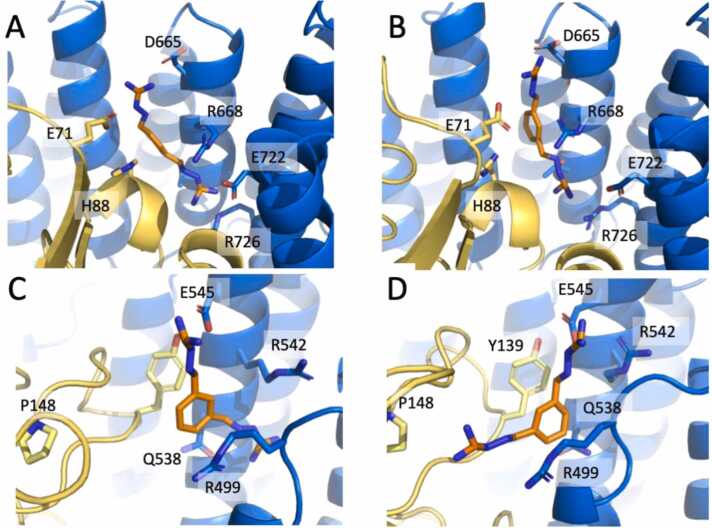
Prevalent conformations of **2a**^**+**^ in *site1* and *site3*. A, B *site1* with **2a**^**+**^ (sticks with orange carbon atoms) in the less occupied conformation (28.5% of the simulation time) and in the stable conformation (67.3%), respectively (VPS29 and VPS35 are represented as yellow and blue cartoons with selected residues as sticks). C and D *site3* with **2a**^**+**^ in the less occupied (14.8%) and stable (71.1%) conformation, respectively.

To further study the behavior of the ligand in the three binding sites, we decided to perform an additional MD simulation with the ligand in the single charged state (**2a**^**+**^) for 1 µs. During this simulation the qualitative behavior of the ligand was similar to that observed for the uncharged molecule, i.e. with higher binding stability for *site1* and *site3*. Cluster analysis showed **2a**^**+**^ maintaining two stable conformations in *site1*: the first from 4.0 ns to ∼330 ns (28.5%) with transitions to the second conformation (starting from ∼220 ns) that lasted until the end of the simulations (67.3% of the simulation time). The two conformations of **2a**^**+**^ were quite similar (Fig. 2, A and B), with the phenyl ring located at about 5 Å from the crystallographic configuration due to the loop-to-helix transition of Gly87-Val90 in VPS29.

In *site3,* **2a**^**+**^ adopts a stable conformation from 24 ns to 932.8 ns (71.1%; Fig. 2C) with some transitions (starting from 664.8 ns) to a less occupied conformation (14.8%; Fig. 2D). In both conformations the interaction is stabilized by a salt bridge with VPS35 Glu545.

## 4 Conclusion

With the attempt to provide new treatments for ALS, R55 was discovered as a pharmacological chaperone able to enhance retromer stability and functionality [13]. The proposed R55 binding site (named *site3* in this work) was used to identify compound **2a** [15], acting as stabilizer of VPS29/VPS35 interface. In this work, from the analysis of two protein crystals of the VPS35sh/VPS29 complex in the presence of **2a**, two additional binding sites were identified (named *site1* and *site2*). Due to the poor electron density of some portions of the ligand, to further characterize the three binding sites, we decided to perform computational analysis by MD simulation.

The three binding sites were firstly analyzed with 18 MD simulations of 100 ns each, run in the presence of three **2a** compounds, either neutral (nine simulations) or fully charged **2a**^**+2**^ (nine simulations), located in front of the three identified binding sites. Our results showed that charged **2a**^**+2**^ behaves like a promiscuous ligand, able to bind transiently to different sites on the VPS35/VPS29 interface with preferential stability in *site2*. *Site2* is mainly located in a small superficial cleft of VPS35 between the extremities of four contiguous alpha helices. Such binding site seems to be incompatible with the stabilization of the VPS35/VPS29 interface, being mostly located on VPS35 and involving only transiently VPS29. It is worth noting that the poor stabilizing effect of **2a**^**+2**^ in *site2* could be enhanced by elongating one of the 2 guanyl hydrazone arms to strengthen the interaction with VPS29.

In contrast, the uncharged or single charged molecule **2a** can stably interact with either *site1* or *3*, showing that they are both potentially useful for the stabilization of the complex. Cluster analysis of **2a**^**+**^ in *site1* showed two similar conformations of the ligand maintained for most of the simulations time (∼96%; Fig. 2, A and B) with higher stability of the buried guanyl hydrazone arm as already observed in the crystal structure.

In contrast, the two stable conformations of **2a**^**+**^ in *site3* (85.9% of the simulation time) were quite different (Fig. 2, C and D). In *site3*, the charged guanyl hydrazone arm was stabilized by the interaction with Glu545, and the other arm displayed two different orientations rotated by ∼180 degrees relative to each other: one in contact with VPS29 loop 130–150 (71.1%; Fig. 2C) and the other pointing toward the C-terminal helices of VPS35 (14.8%; Fig. 2D).

In conclusion, our data supported the propensity of **2a** to tightly bind to *site3* (R55 binding site) and underlined the importance of the newly identified *site1* for the development and optimization of new molecular chaperones for the stabilization of the retromer complex.

## Funding

Fondazione AriSLA - Fondazione Italiana di Ricerca per la Sclerosi Laterale Amiotrofica (https://www.arisla.org), Title of the project: “*TRAILER, Therapeutic effects of retromer stabilization in Amyotrophic Lateral Sclerosis*” (FG_25/2019).

Italian National Recovery and Resilience Plan (NRRP), M4C2, funded by the European Union - NextGenerationEU (Project IR0000011, CUP B51E22000150006, “EBRAINS-Italy”).
