# Supplementary material for: Stabilization of the retromer complex: Analysis of novel binding sites of bis-1,3-phenyl guanylhydrazone 2a to the VPS29/VPS35 interface
Source: Comput Struct Biotechnol J. 2024 Mar 2;23:1088–93. doi: 10.1016/j.csbj.2024.02.026 (PMC10937258; doi:10.1016/j.csbj.2024.02.026)
Supplement: Supplementary file 1 — Supplementary material [file mmc1.docx]

**Supplemental materials for the paper:**

**Stabilization of the retromer complex: analysis of novel binding sites of bis-1,3-phenyl guanylhydrazone 2a to the VPS29/VPS35 interface.**

Elisa Fagnani, Francesco Boni, Pierfausto Seneci, Davide Gornati, Luca Muzio^,^ Eloise Mastrangelo, Mario Milani


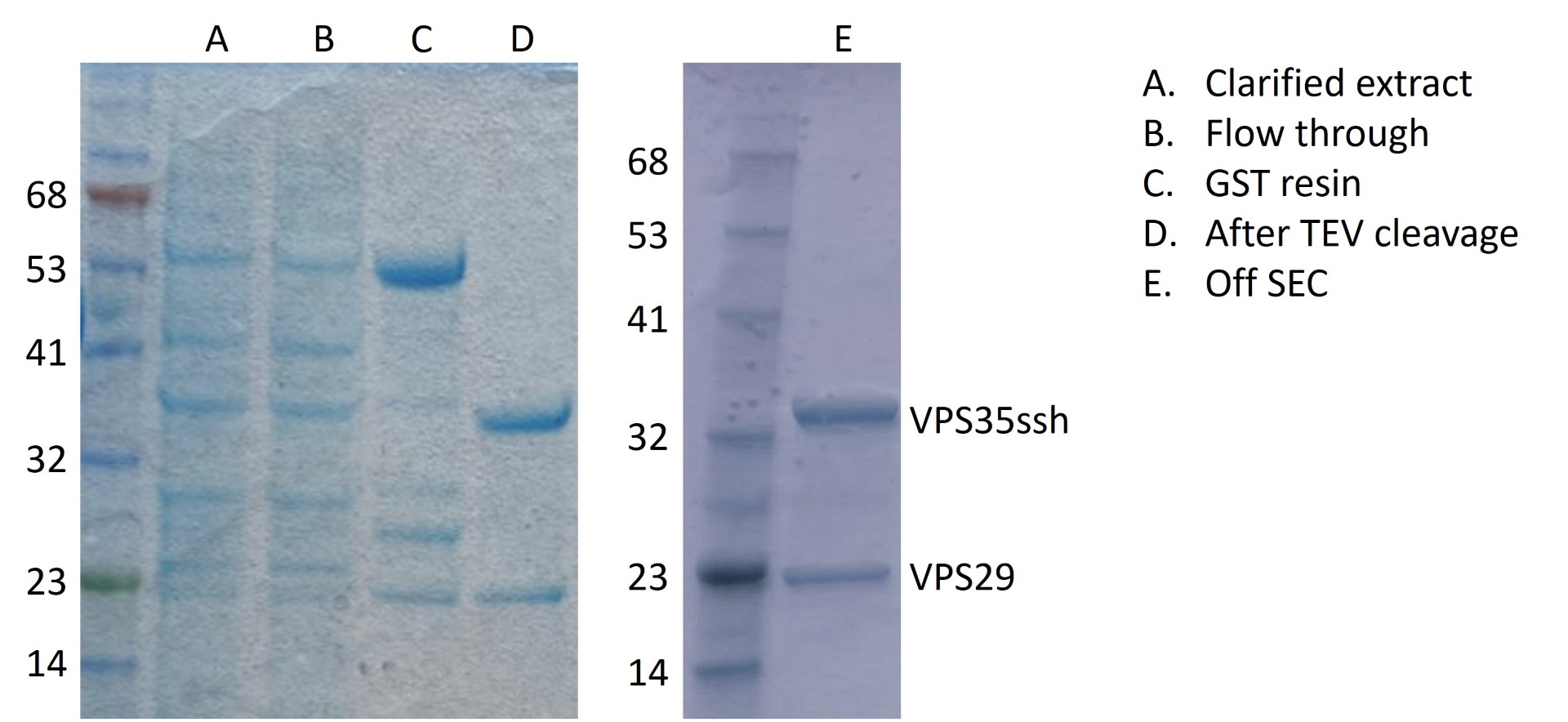


**Figure S1. Purification of the VPS29/VPS35sh complex.** SDS-PAGE gel lines A to D: purification of VPS35sh with GST resin (A-C) and TEV cleavage (D); line E: analysis of the VPS29/VPS35sh complex after SEC.


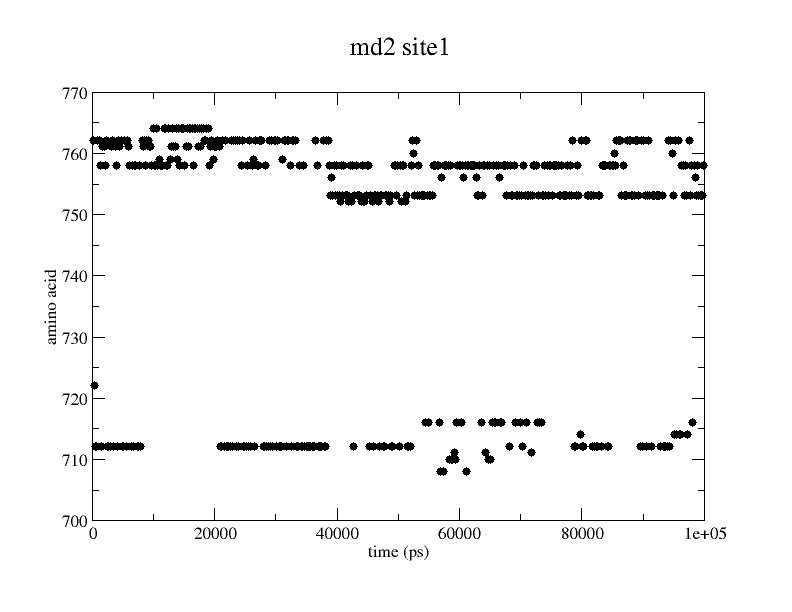

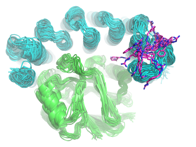


**
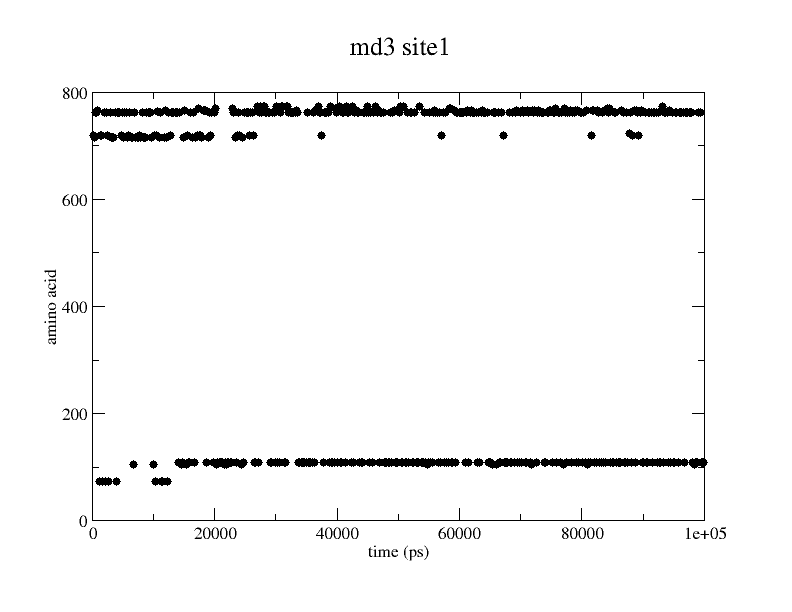

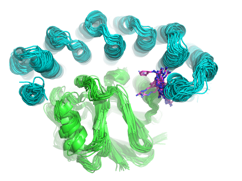

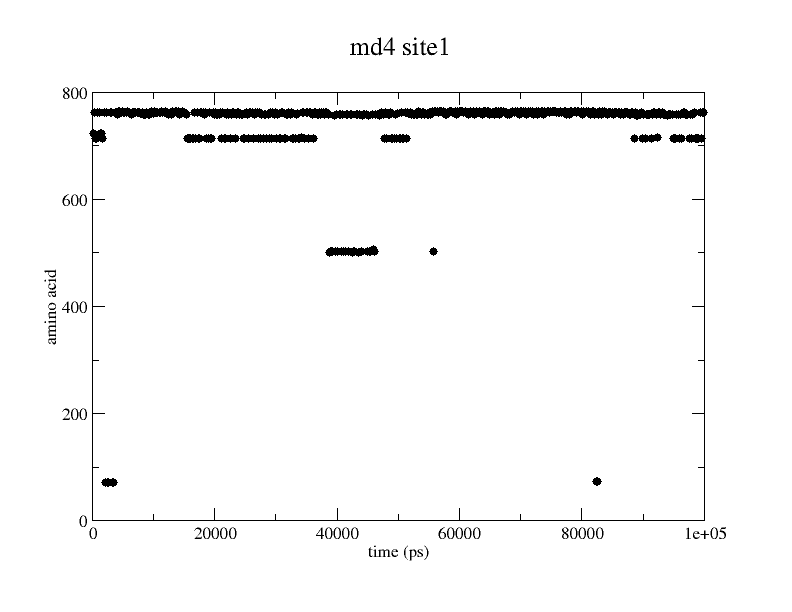

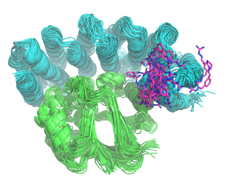

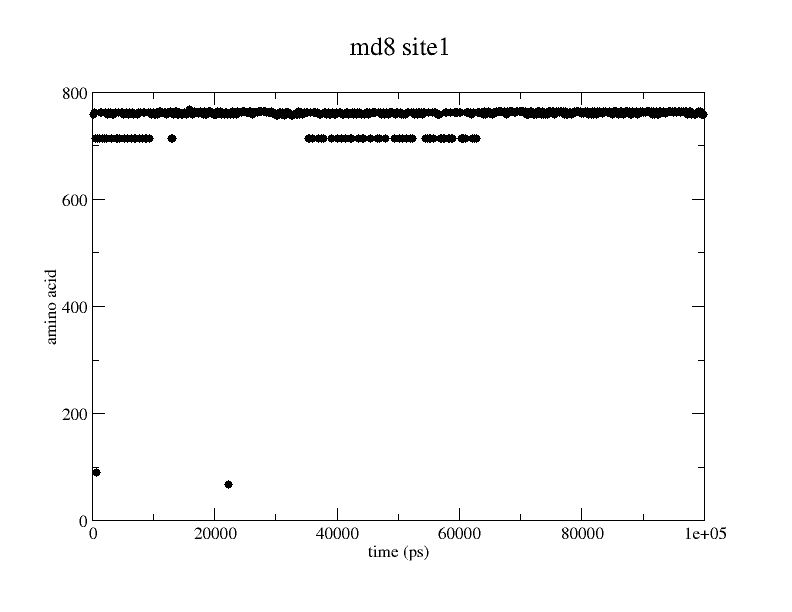

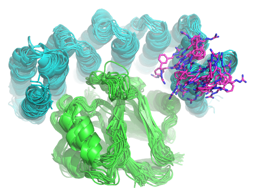
**

**
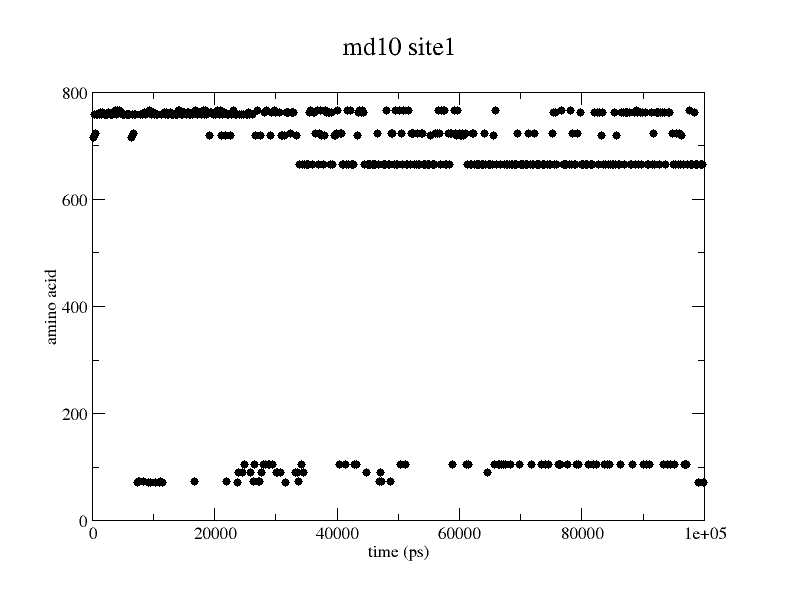

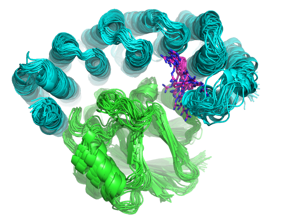

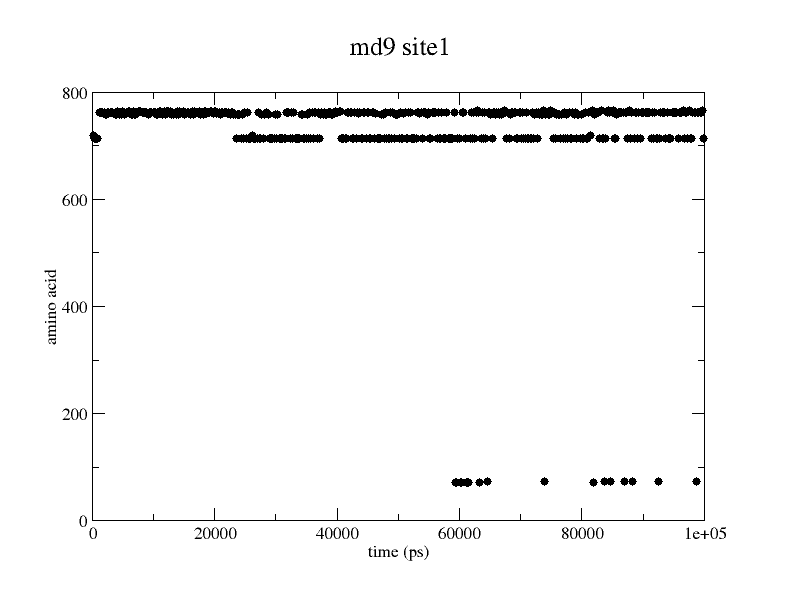

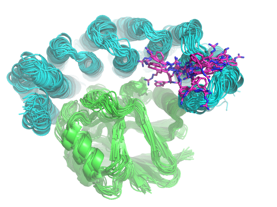

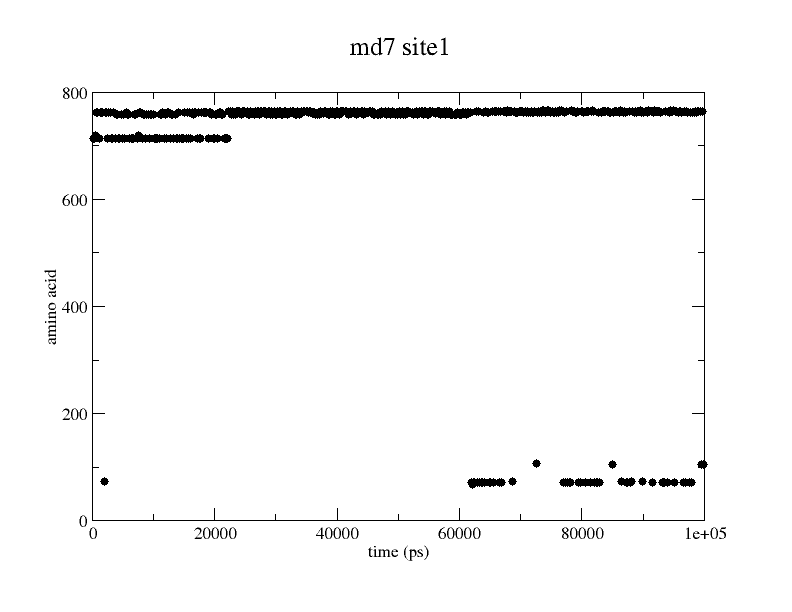

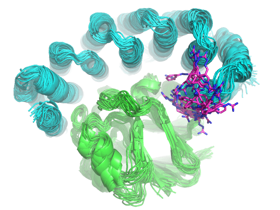

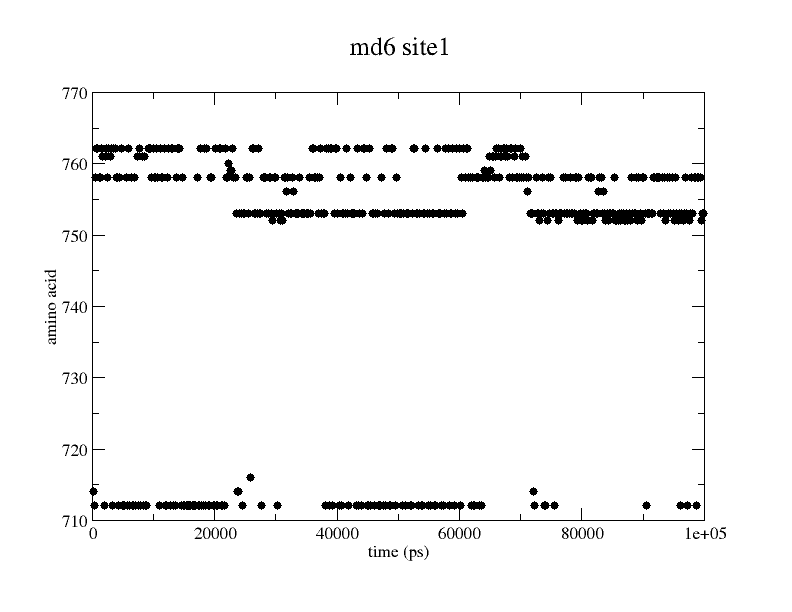

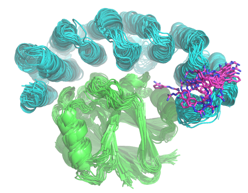

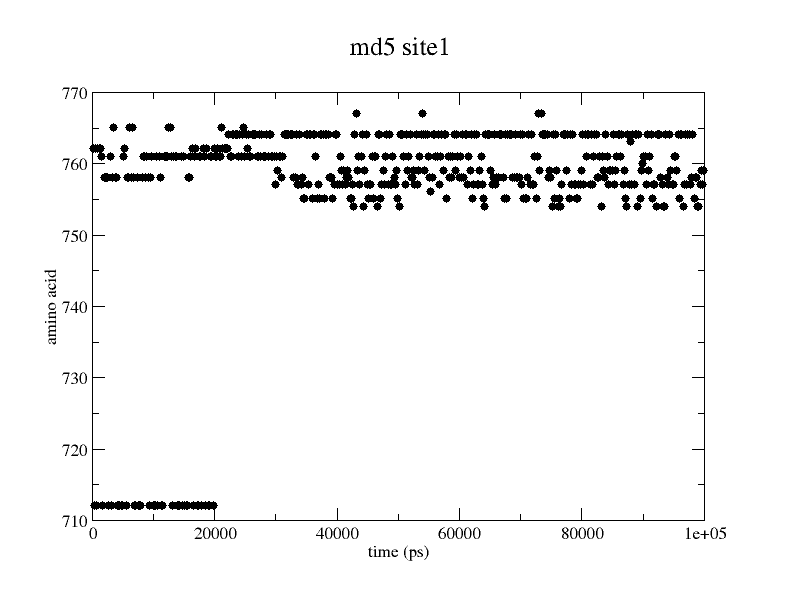

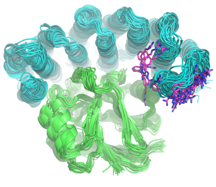
**

**Figure S2. MD simulations of 2a^2+^ in *site1*.** Each of the 9 graphs reports in the ordinate the amino acid (identified with its number in the sequence) with the lower distance from **2a^2+^** in *site1* during each simulation. The insets show the superpositions of 21 structures of VPS29/VPS35 (green/cyan cartoons) taken every 5 ns (from 0 to 100 ns) together with **2a^2+^** (sticks with pink carbon atoms).


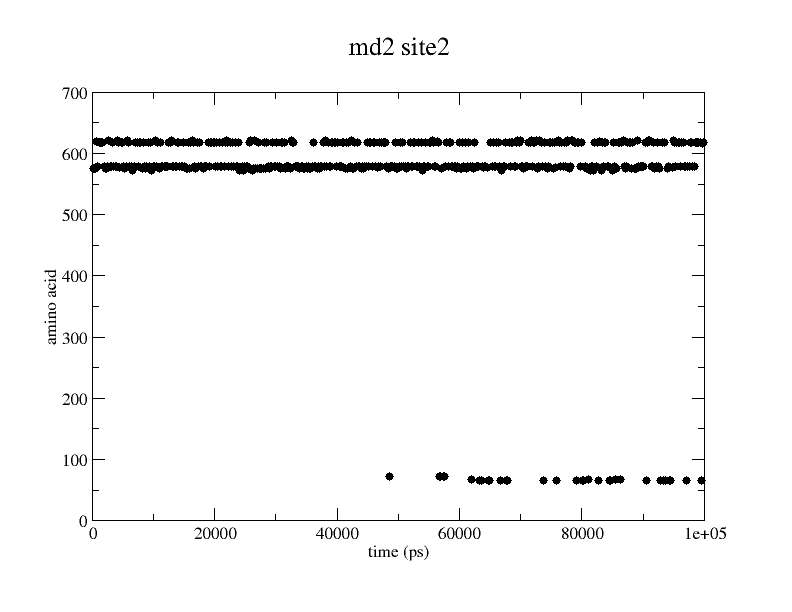

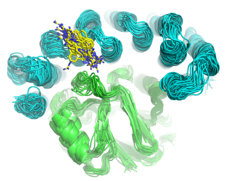

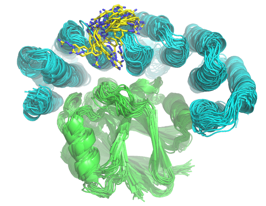

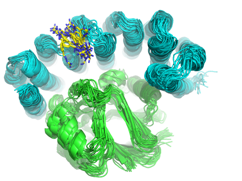
site2


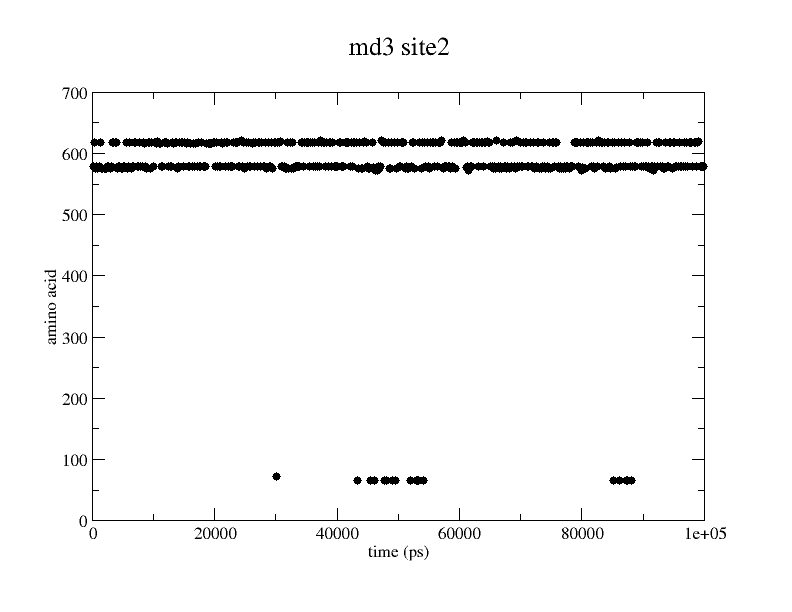

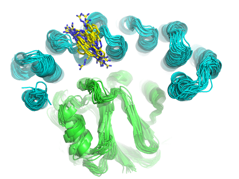

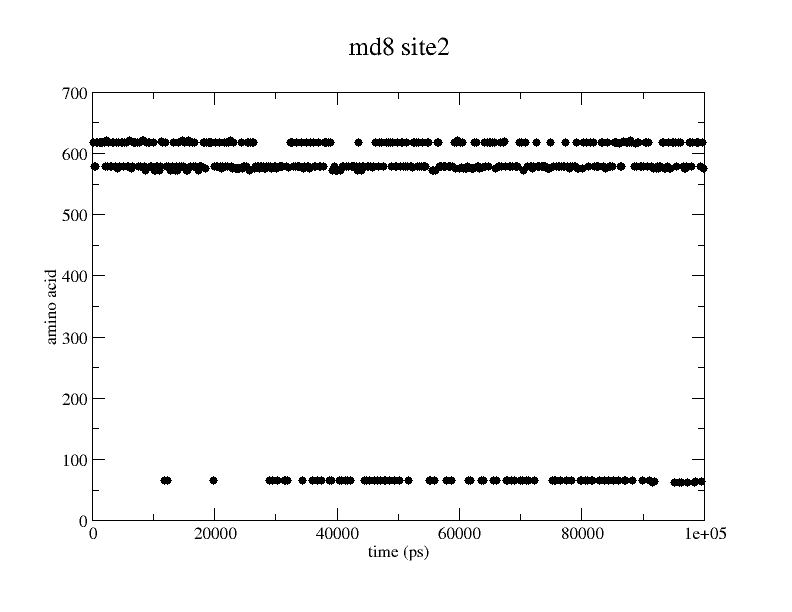

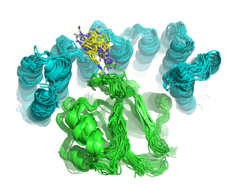

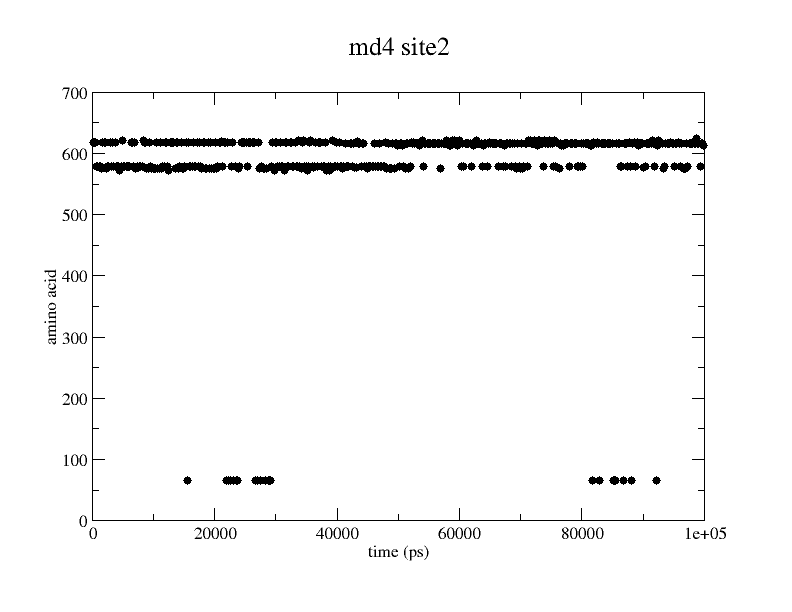


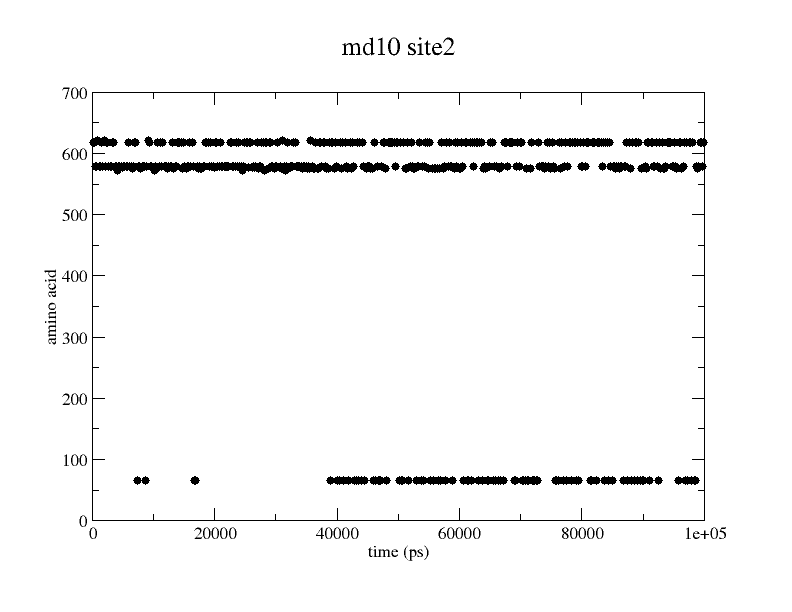

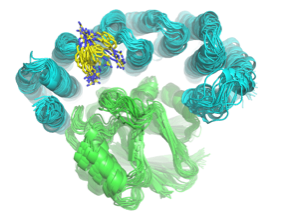

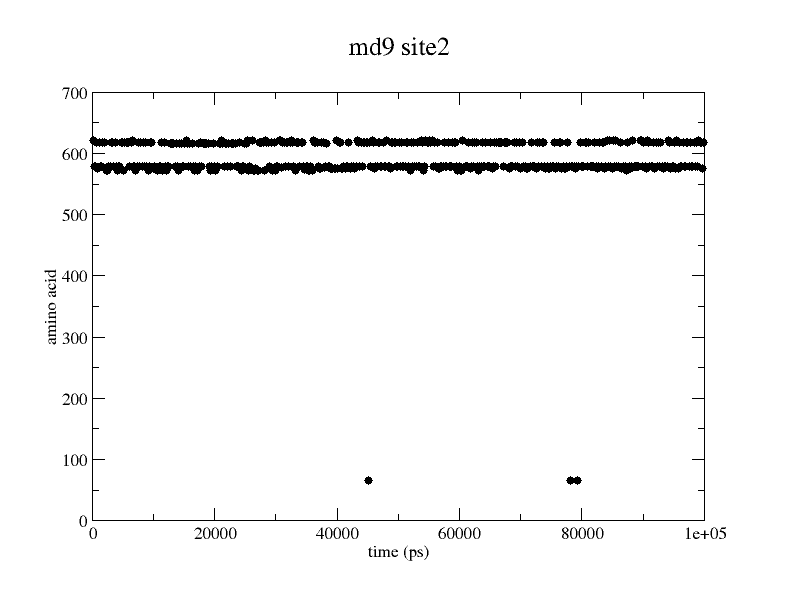

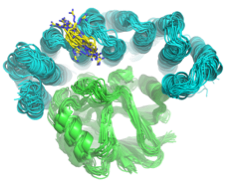

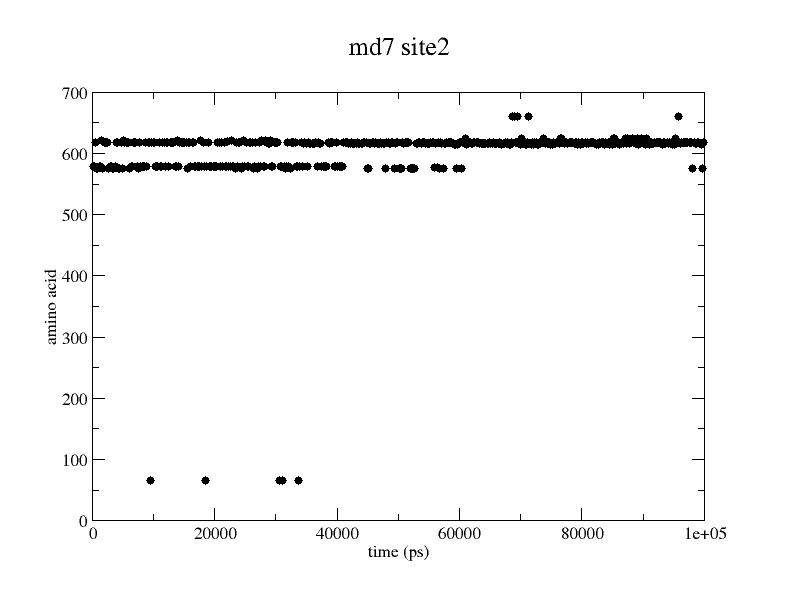

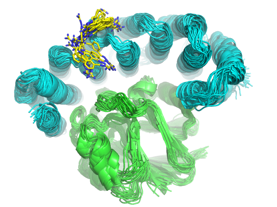

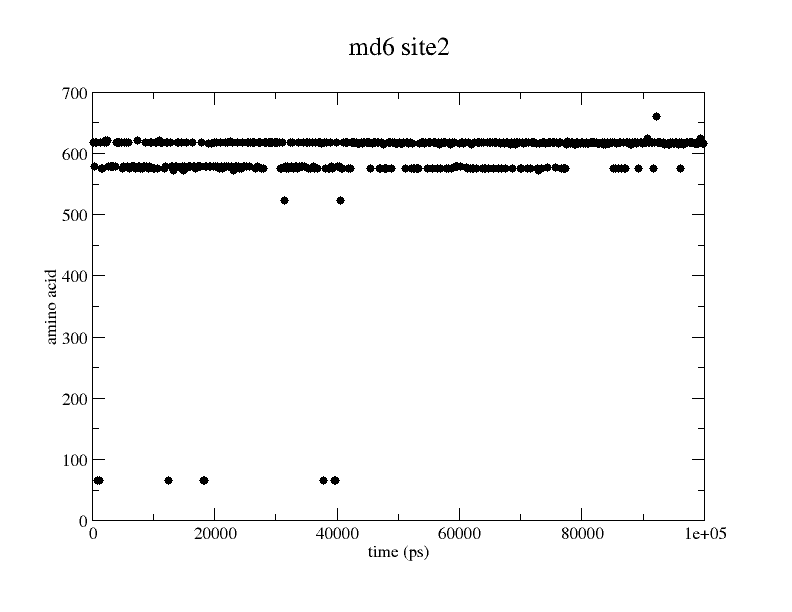
**
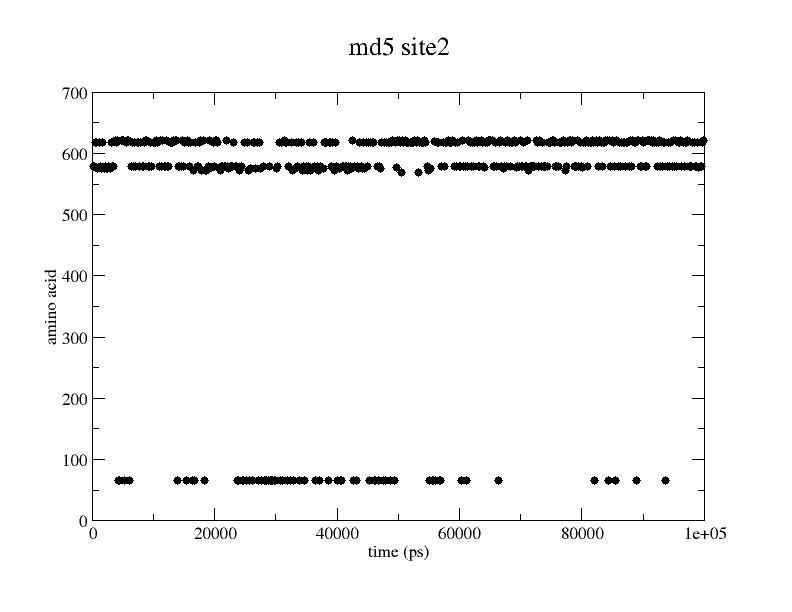

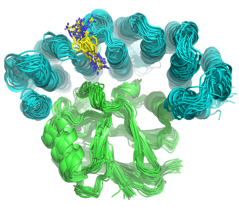
**

**Figure S3. MD simulations of 2a^2+^ in *site 2*.** Each of the 9 graphs report in the ordinate the amino acid (identified with its number in the sequence) with the lower distance from **2a^2+^** in *site2* during each simulation. The insets show the superpositions of 21 structures of VPS29/VPS35 (green/cyan cartoons) taken every 5 ns (from 0 to 100 ns) together with **2a^2+^** (sticks with yellow carbon atoms).


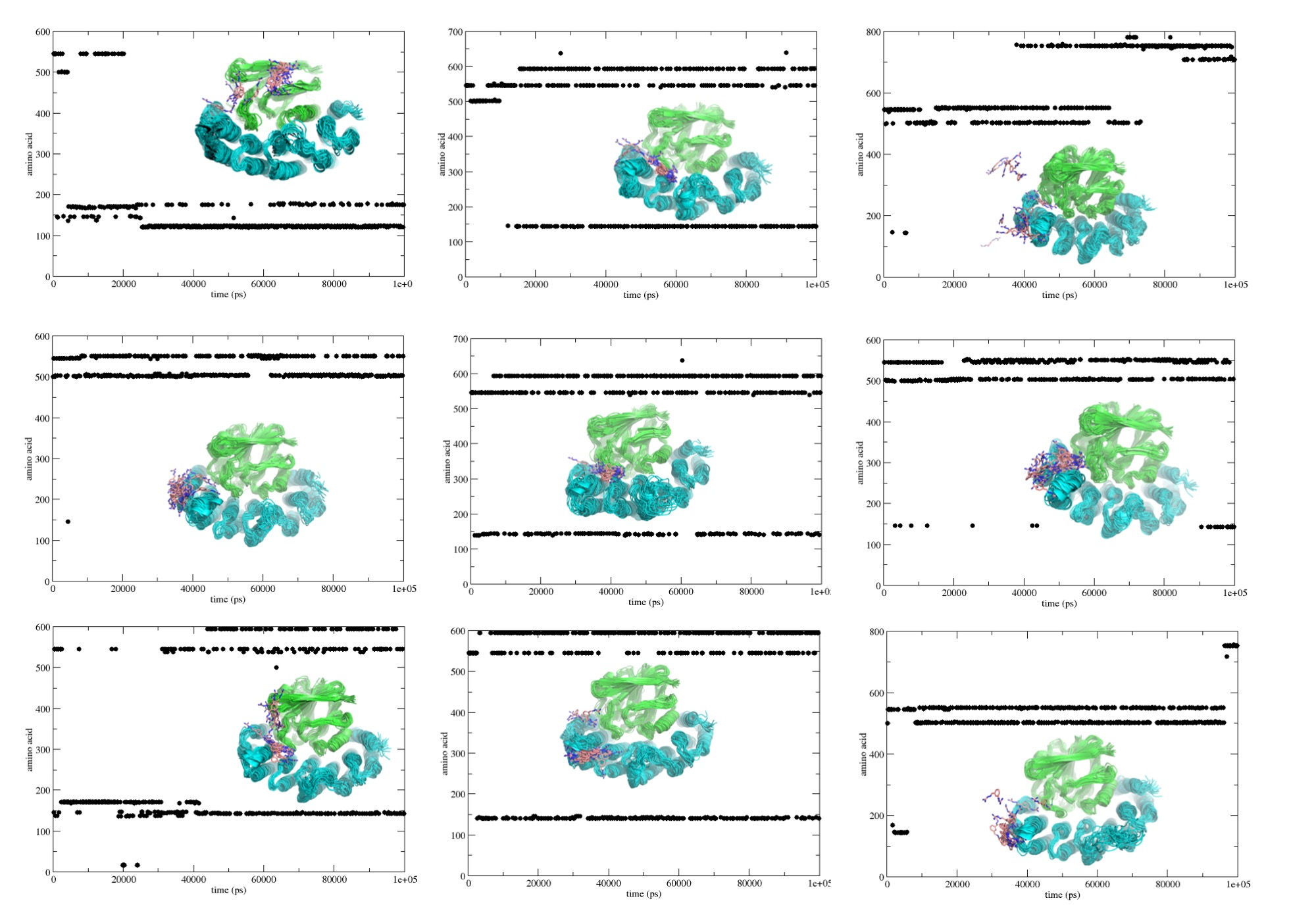


**Figure S4. MD simulations of 2a^2+^ in *site3*.** Each of the 9 graphs reports in the ordinate the amino acid (identified with its number in the sequence) with the lower distance from **2a^2+^** in *site3* during each simulation. The insets show the superpositions of 21 structures of VPS29/VPS35 (green/cyan cartoons) taken every 5 ns (from 0 to 100 ns) together with **2a^2+^** (sticks with pink carbon atoms).


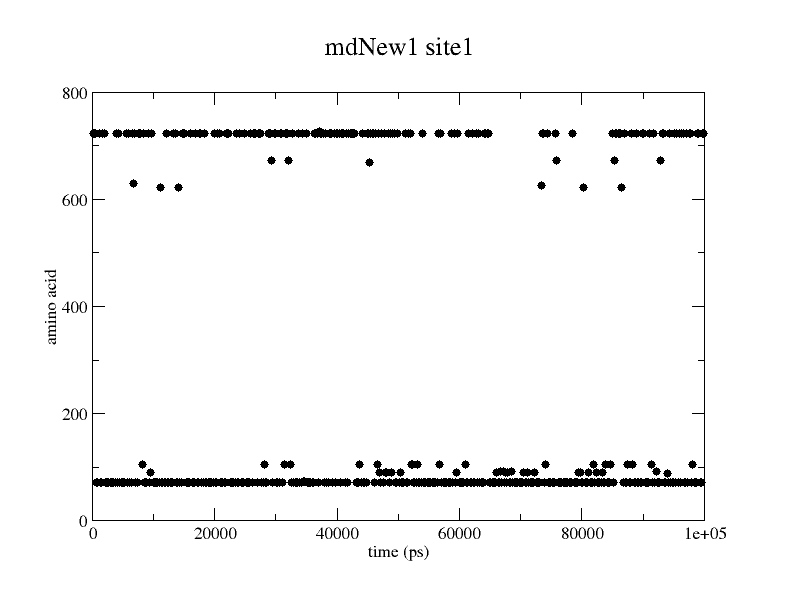

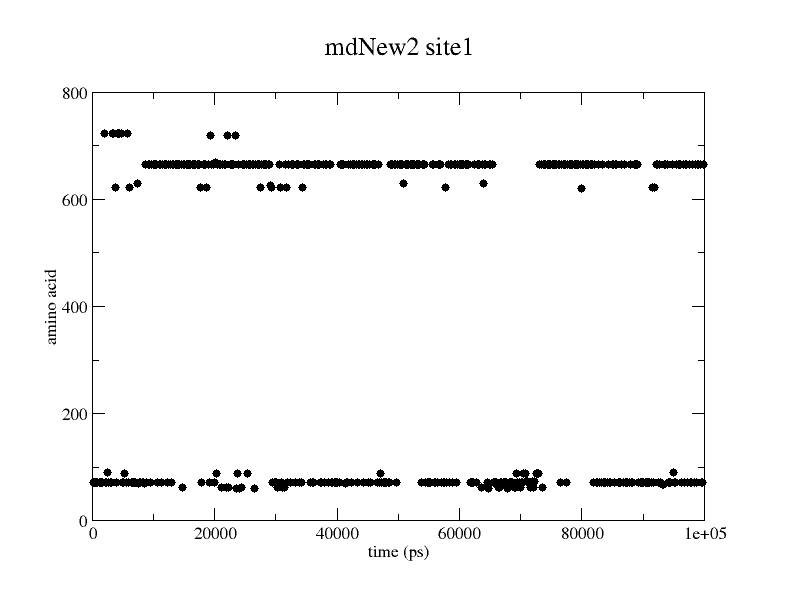

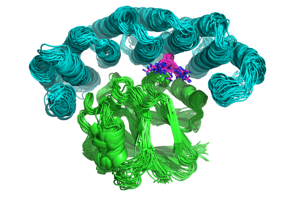

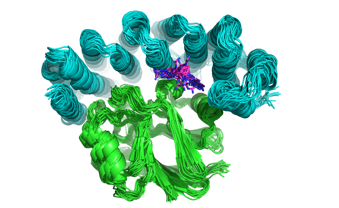

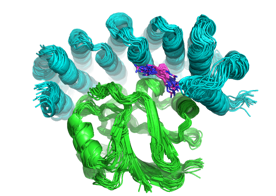

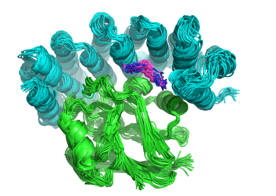

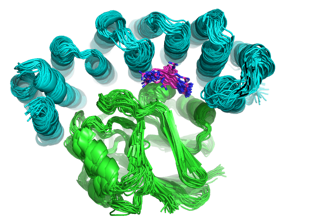

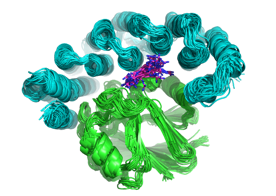


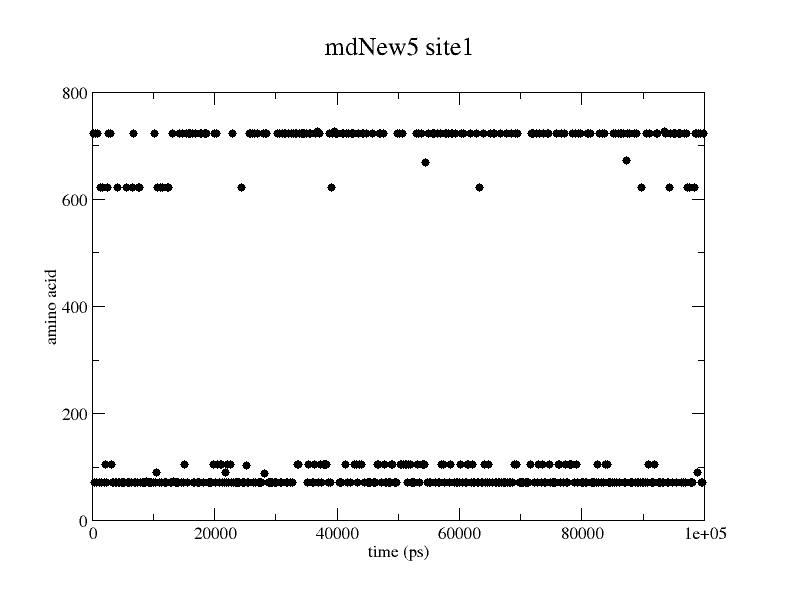

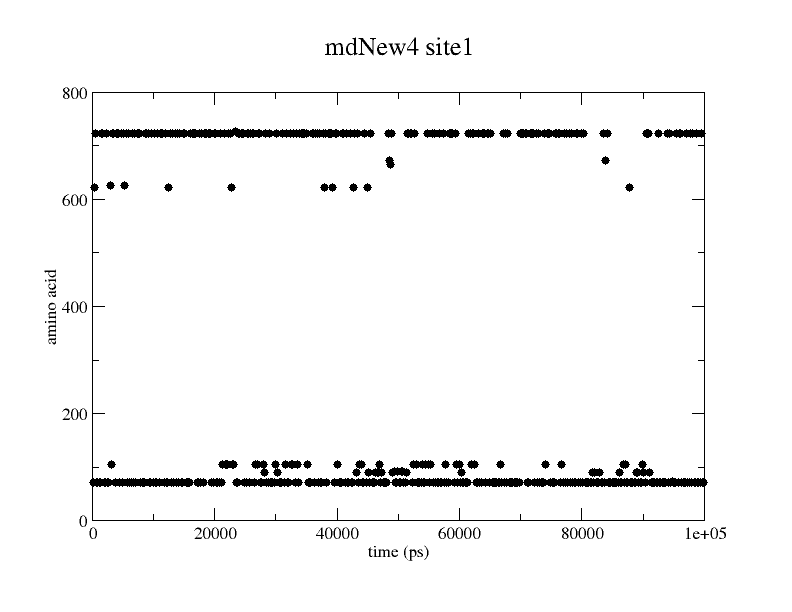

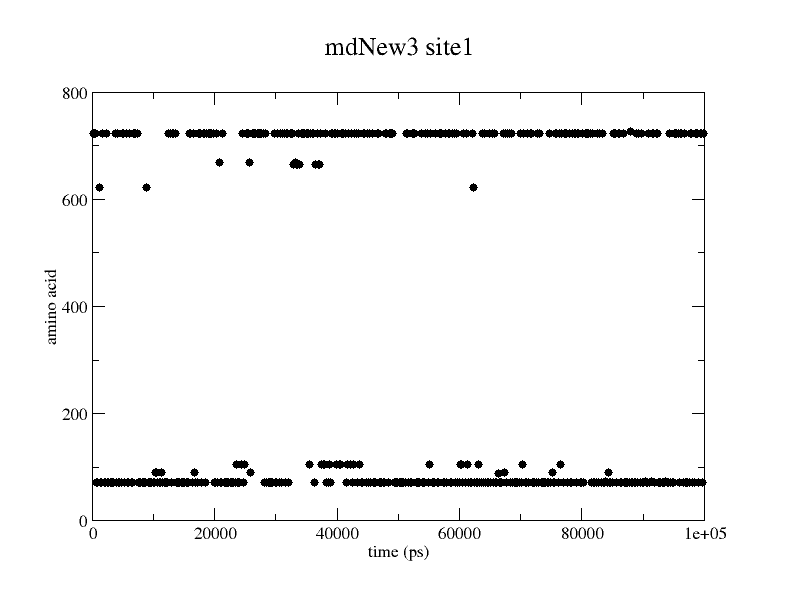

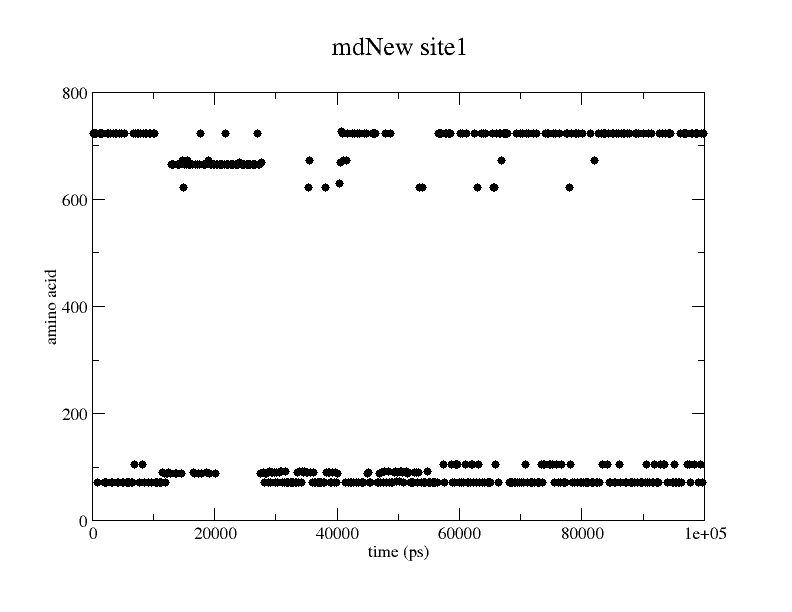


**
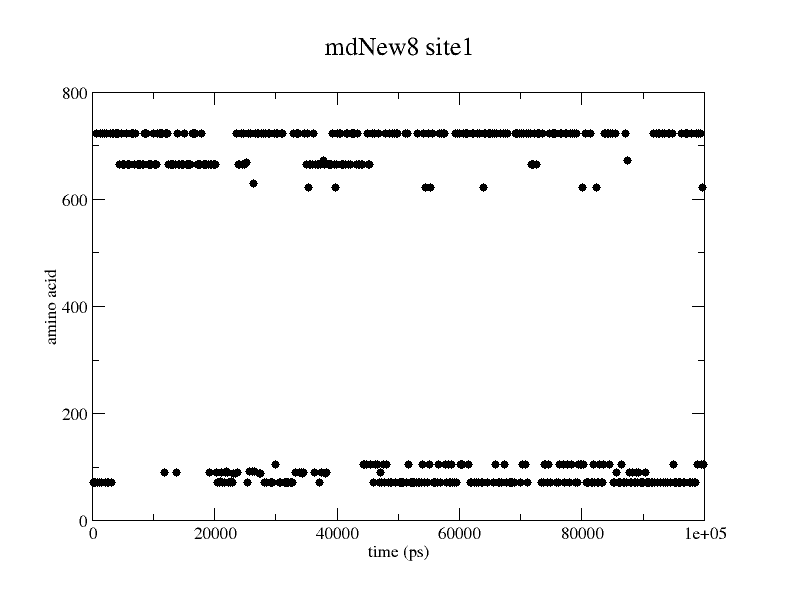

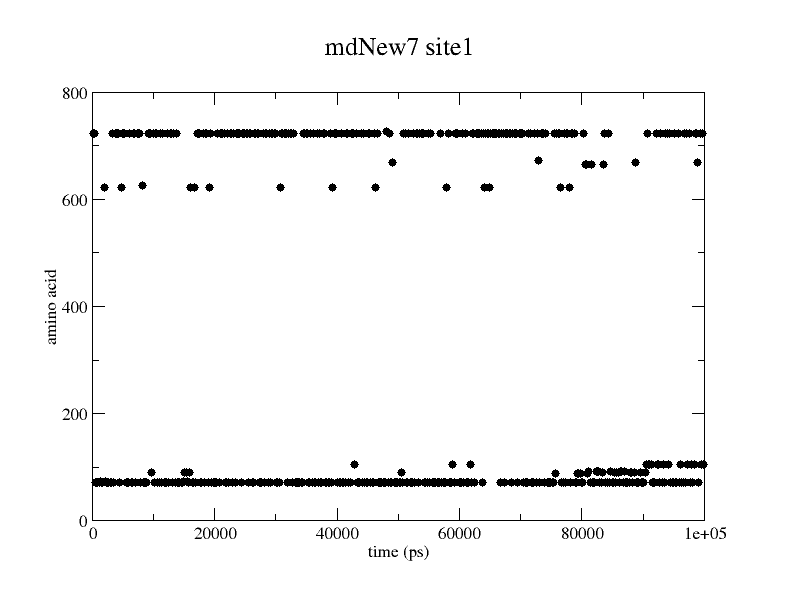

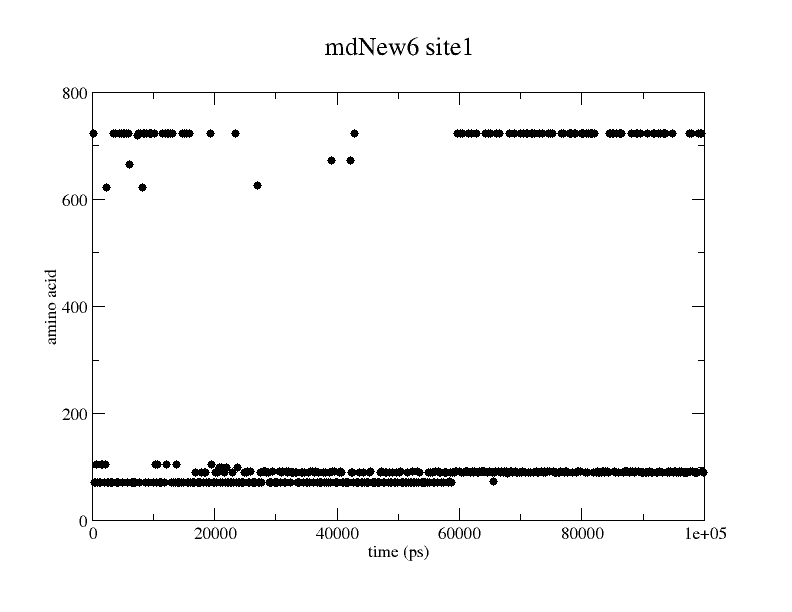

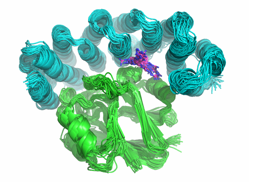

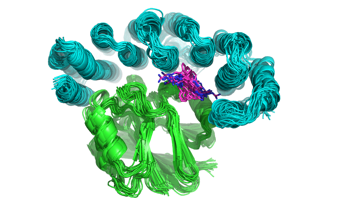

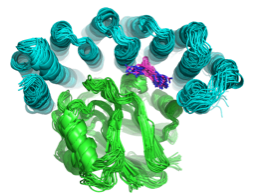
**

**Figure S5. MD simulations of 2a in *site1*.** Each of the 9 graphs reports in the ordinate the amino acid (identified with its number in the sequence) with the lower distance from **2a** in *site1* during each simulation. The insets show the superpositions of 21 structures of VPS29/VPS35 (green/cyan cartoons) taken every 5 ns (from 0 to 100 ns) together with **2a** (sticks with pink carbon atoms).


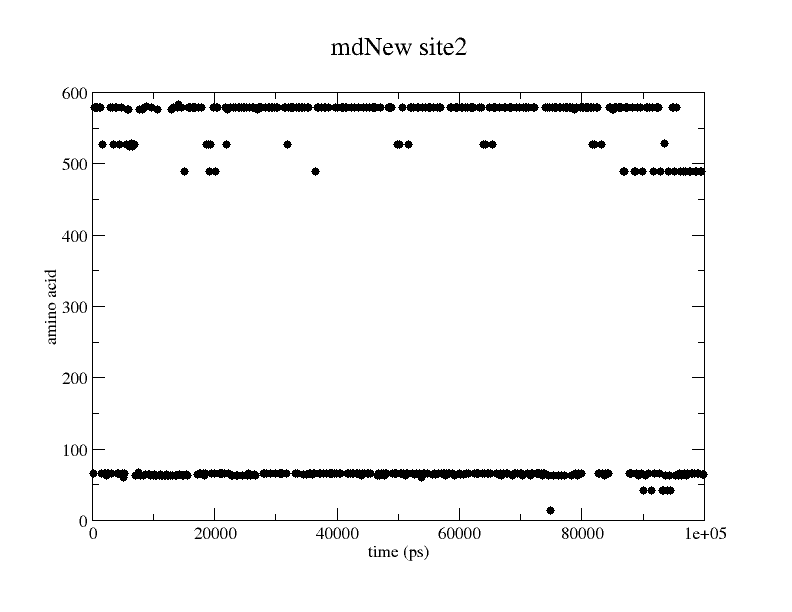

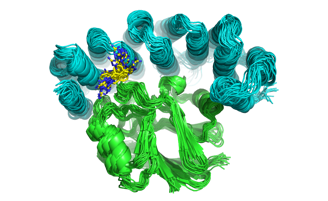


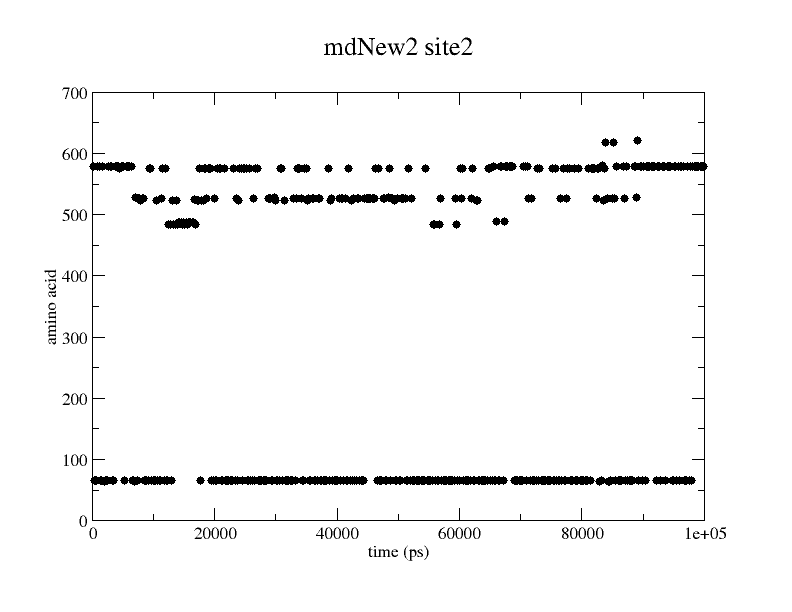

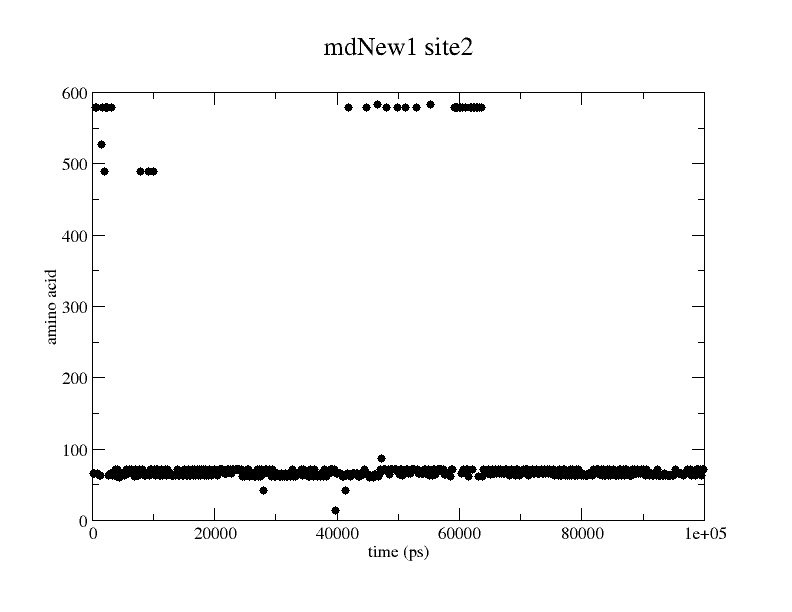

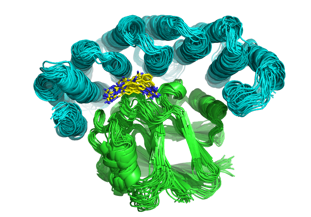

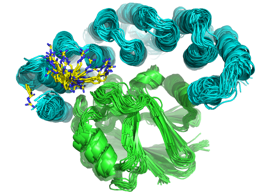


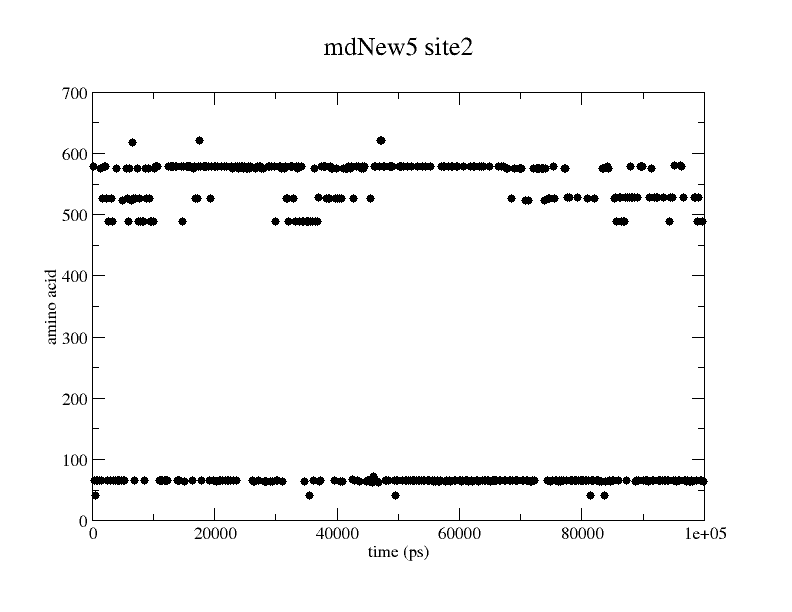

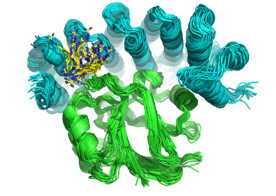

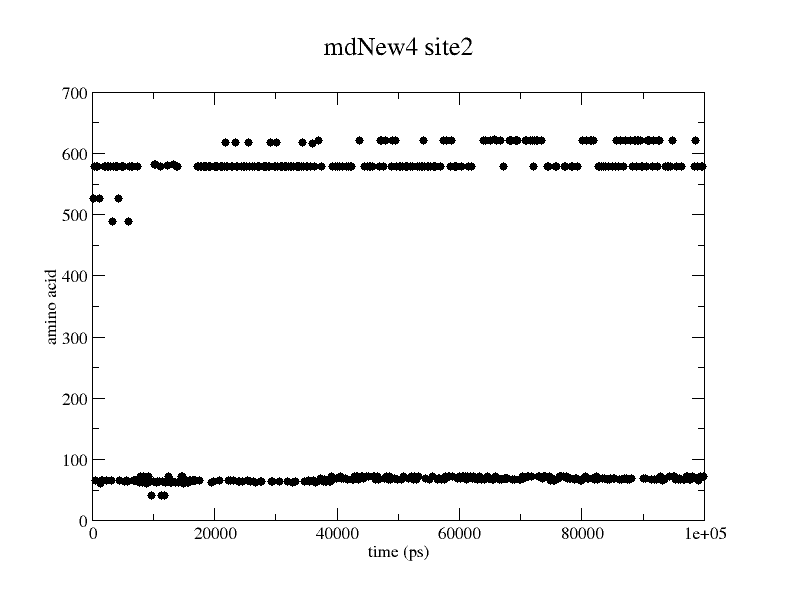

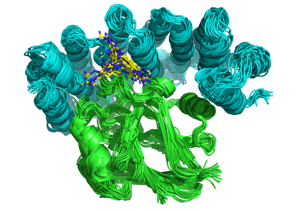

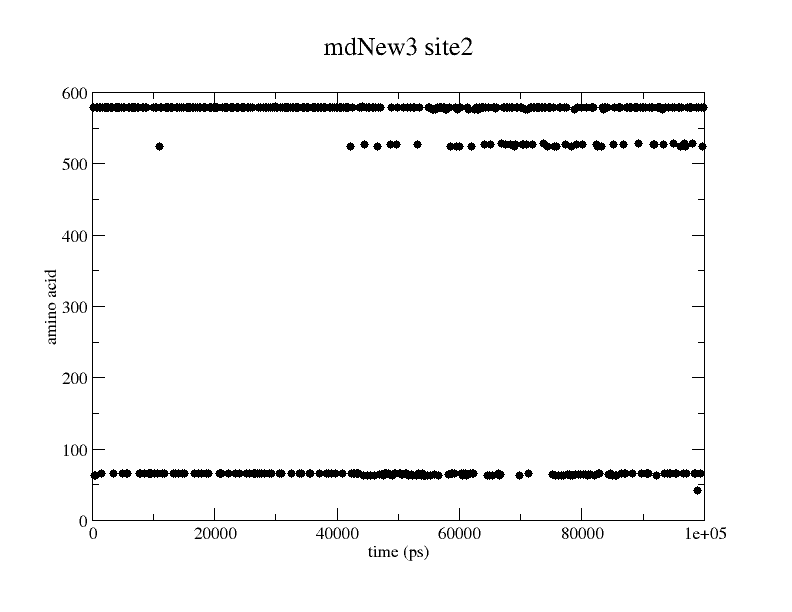

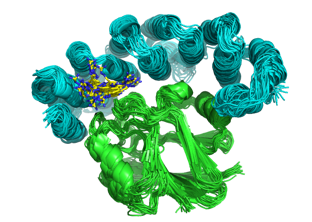

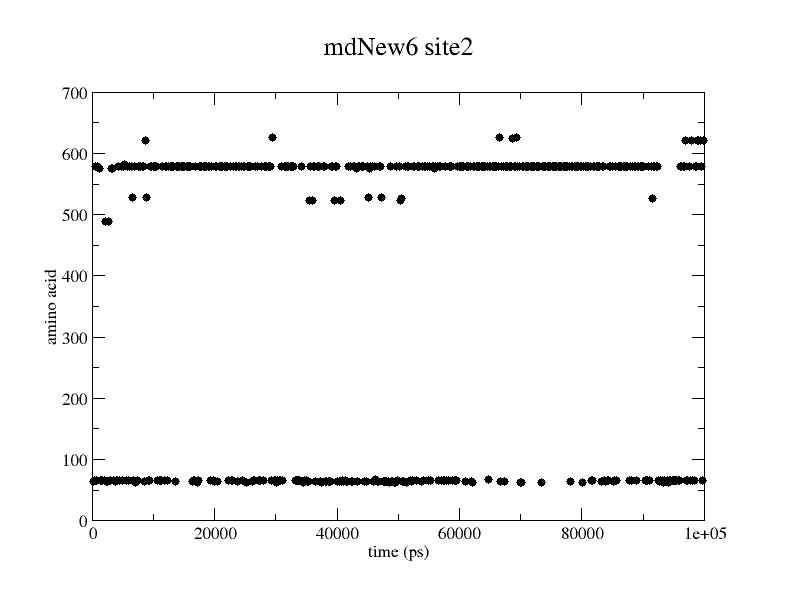

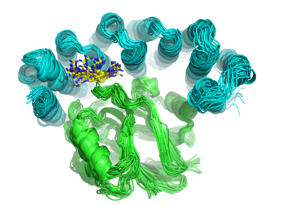

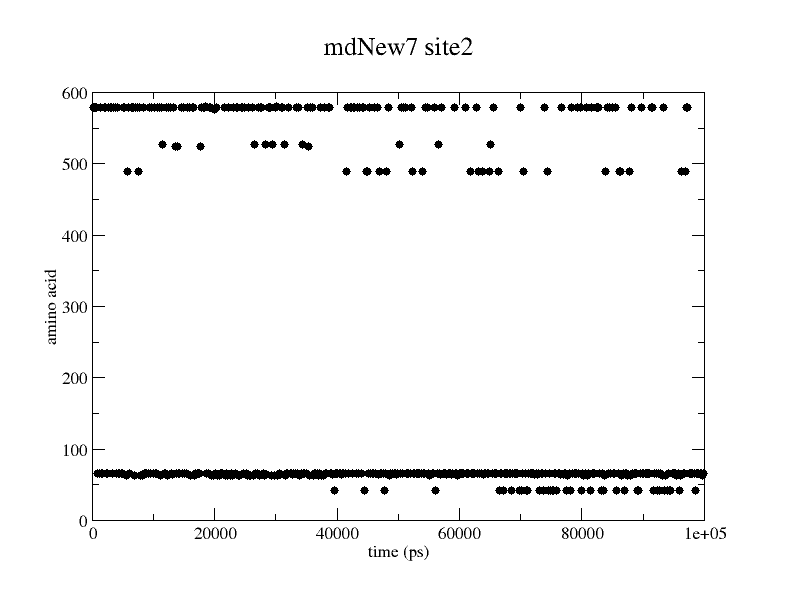

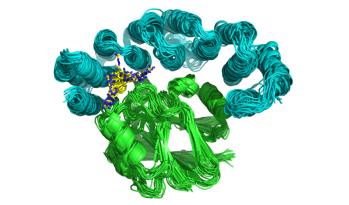

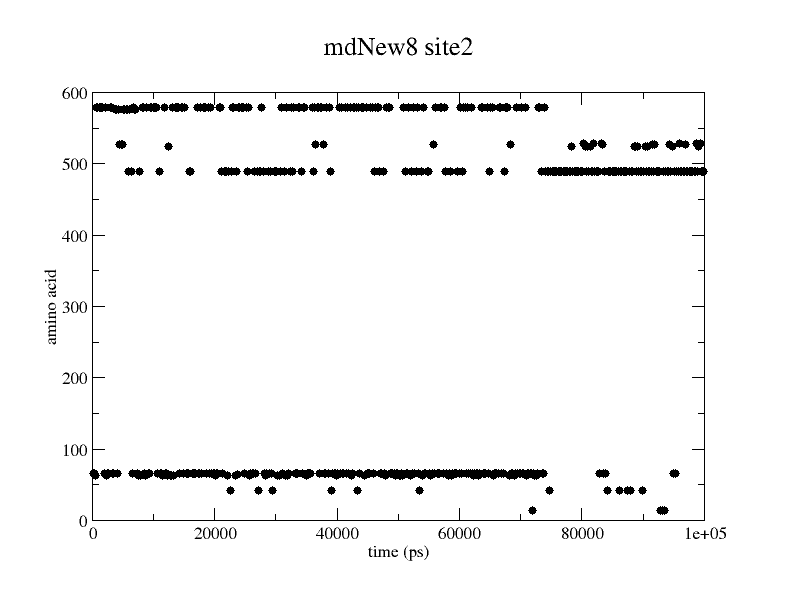

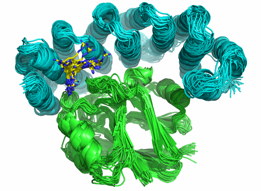


**Figure S6. MD simulations of 2a in *site2*.** Each of the 9 graphs reports in the ordinate the amino acid (identified with its number in the sequence) with the lower distance from **2a** in *site2* during each simulation. The insets show the superpositions of 21 structures of VPS29/VPS35 (green/cyan cartoons) taken every 5 ns (from 0 to 100 ns) together with **2a** (sticks with yellow carbon atoms).


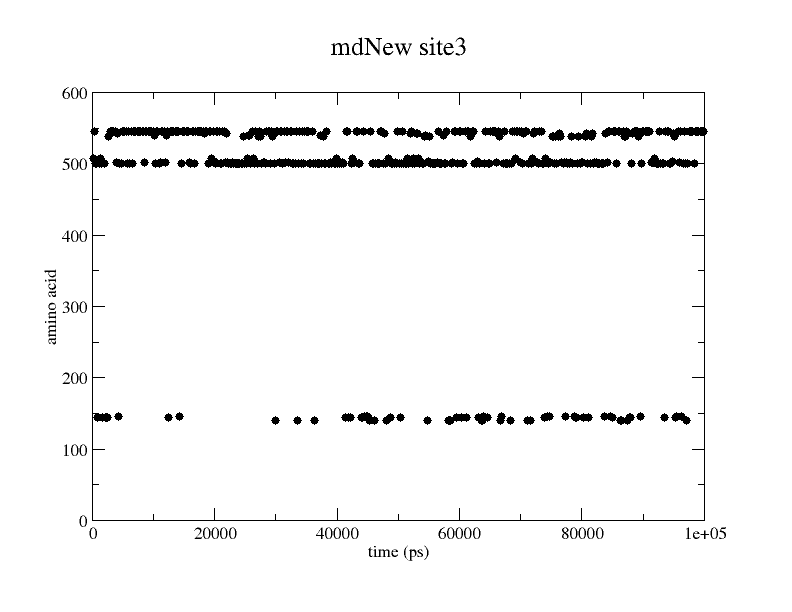

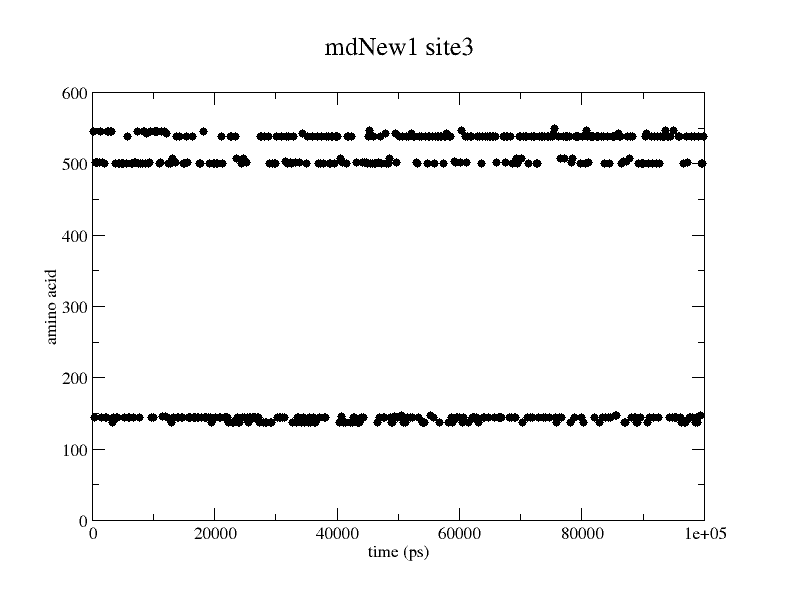

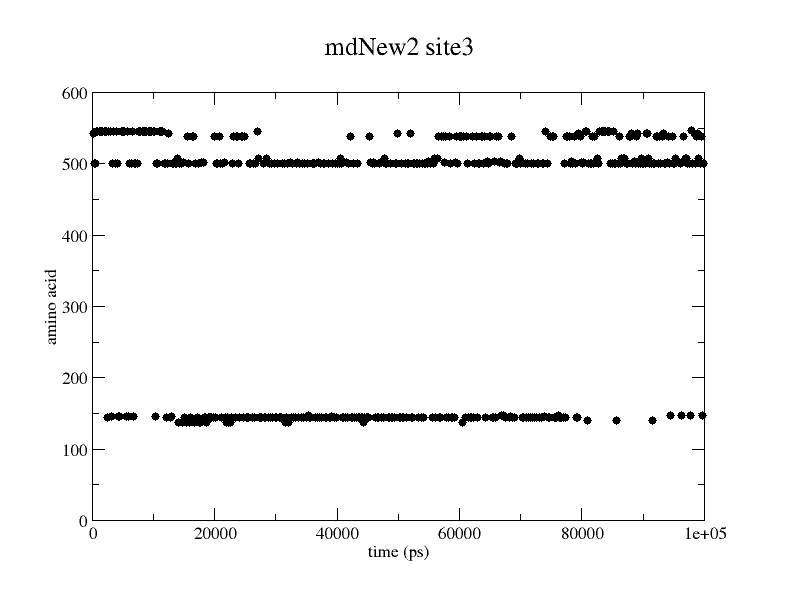

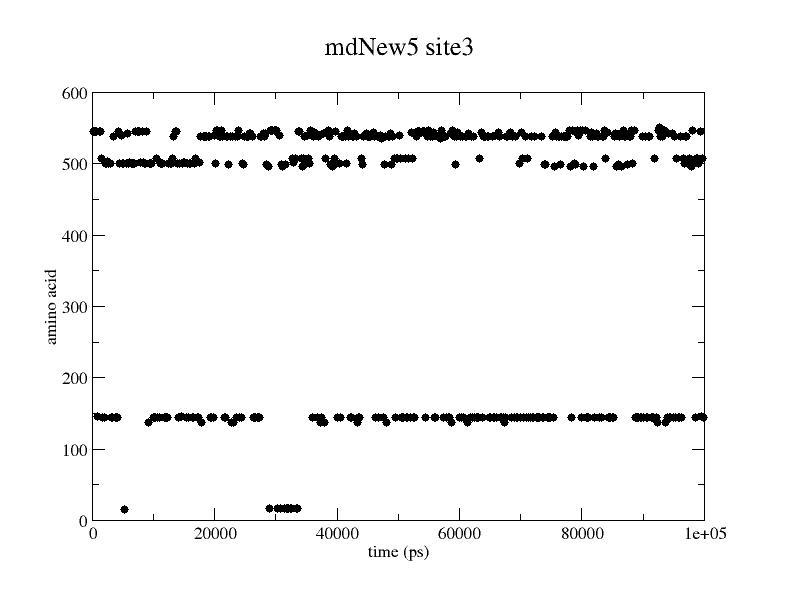

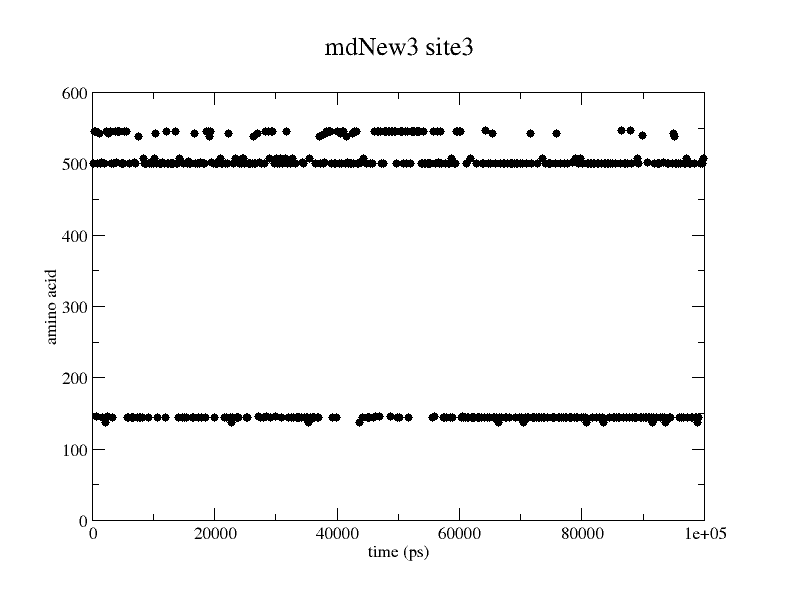

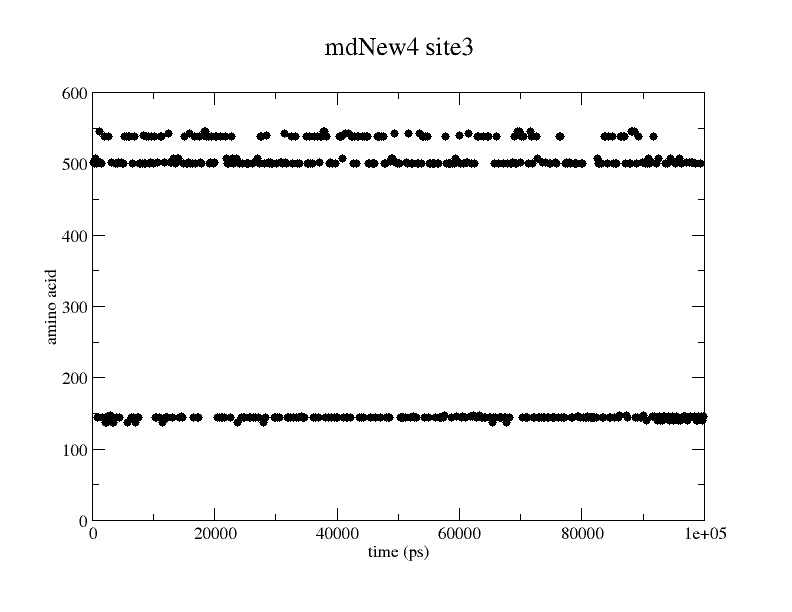

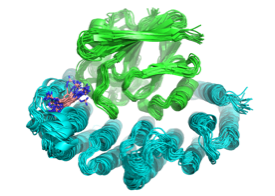

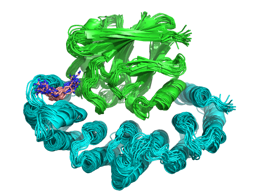

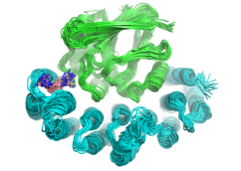

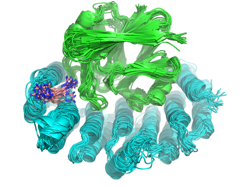

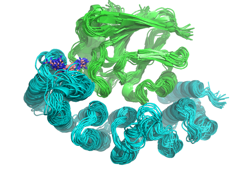

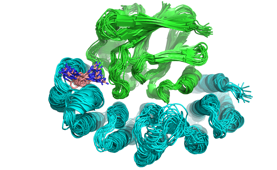


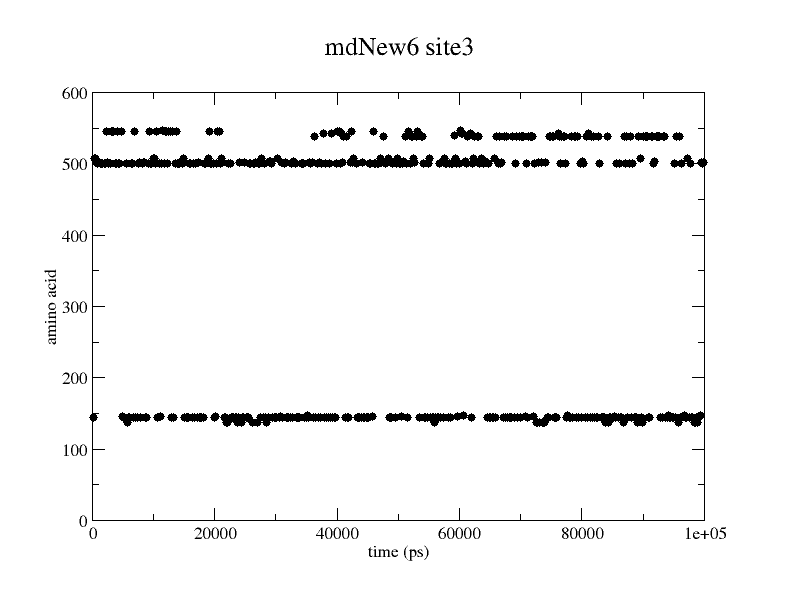

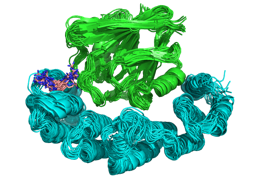

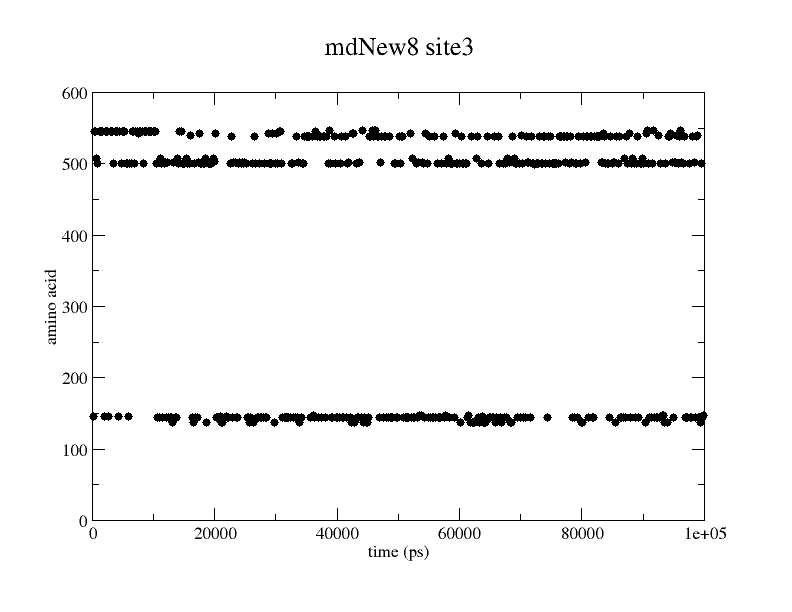

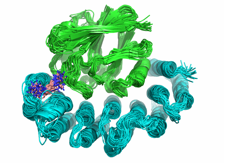

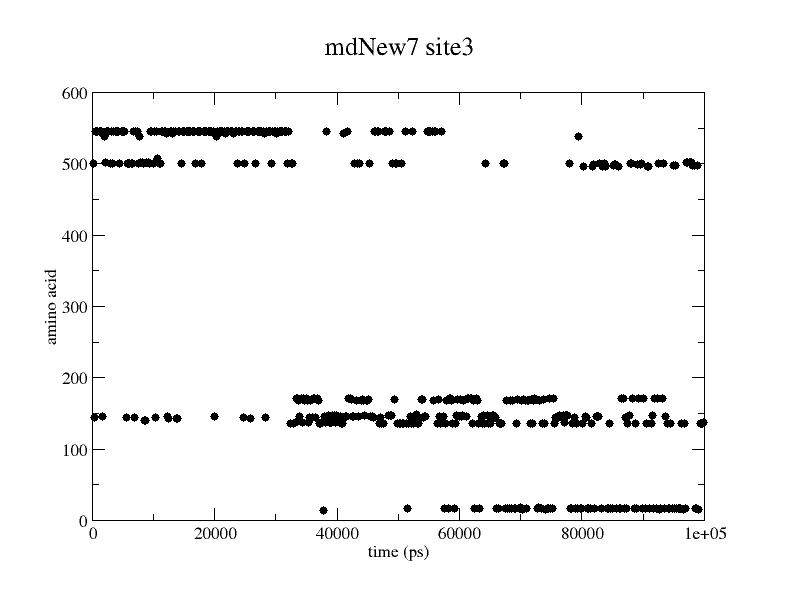

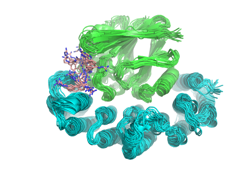


**Figure S7. MD simulations of 2a in *site3*.** Each of the 9 graphs reports in the ordinate the amino acid (identified with its number in the sequence) with the lower distance from **2a** in *site3* during each simulation. The insets show the superpositions of 21 structures of VPS29/VPS35 (green/cyan cartoons) taken every 5 ns (from 0 to 100 ns) together with **2a** (sticks with pink carbon atoms).

**Table S1*.* Amino acids in contact with 2a in *site1* in the most populated structural cluster for each of the nine simulations.**

| **Simulations** | 1 | 2 | 3 | 4 | 5 | 6 | 7 | 8 | 9 |  |
| --- | --- | --- | --- | --- | --- | --- | --- | --- | --- | --- |
| **Reference structure of the cluster [ns]** | 89.0 | 55.6 | 61.0 | 15.4 | 83.6 | 59.2 | 78.0 | 77.2 | 78.8 | **Total number of structures** |
| **Number of structures**  **in each cluster** | 277 | 204 | 326 | 303 | 368 | 465 | 202 | 157 | 289 | = 2591 |
| **Residue in contact with 2a** | **presence of the contact** | | | | | | | | | **relative occurrence of the contact** [%]^&^ |
| Glu71 | ***** | ***** |  | ***** | ***** |  |  | ***** | ***** | 61.7^&^ |
| **His88** |  | ***** | ***** | ***** | ***** | ***** | ***** | ***** | ***** | 89.3 |
| **Gln89** | ***** |  |  | ***** | ***** | ***** | ***** |  | ***** | 73.5 |
| Ile91 | ***** |  |  |  |  |  | ***** |  |  | 18.5 |
| Leu102 |  | ***** |  |  |  |  |  |  |  | 7.9 |
| Gln105 |  |  |  |  |  |  |  |  | ***** | 11.2 |
| **Lys622** | ***** |  | ***** | ***** | ***** | ***** |  | ***** |  | 73.2 |
| Leu625 | ***** | ***** | ***** |  | ***** |  |  | ***** | ***** | 62.6 |
| Ala626 |  |  |  | ***** | ***** | ***** |  | ***** |  | 49.9 |
| **Thr629** | ***** | ***** | ***** |  | ***** | ***** | ***** | ***** | ***** | 88.3 |
| Asp665 |  |  | ***** |  |  |  |  |  |  | 12.6 |
| **Arg668** | ***** | ***** | ***** | ***** | ***** | ***** | ***** | ***** | ***** | 100.0 |
| Thr672 |  |  |  |  | ***** | ***** |  |  |  | 32.1 |
| **Glu722** | ***** | ***** |  | ***** | ***** | ***** | ***** | ***** | ***** | 87.4 |
| Asn725 |  |  |  |  |  |  |  |  | ***** | 11.2 |
| **Arg726** | ***** | ***** |  | ***** | ***** | ***** | ***** |  |  | 81.4 |

^&^ Relative occurrence of the contact: total number of structures in the clusters where the residue is in contact with **2a** divided for the total number of structures in the clusters. For instance, Glu71 is present in 6 clusters (collecting 1598 structures) / over 9 clusters (collecting 2591 structures): 61.7%.

**Table S2*.* Amino acids in contact with 2a in *site3* in the most populated structural cluster for each of the nine simulations**

| **Simulations** | 1 | 2 | 3 | 4 | 5 | 6 | 7 | 8 | 9 |  |
| --- | --- | --- | --- | --- | --- | --- | --- | --- | --- | --- |
| **Reference structure of the cluster [ns]** | 48.0 | 73.6 | 24.2 | 96.2 | 97.0 | 58.8 | 33.8 | 11.6 | 43.6 | **Total number of structures** |
| **Number of structures**  **in each cluster** | 331 | 332 | 355 | 420 | 339 | 305 | 367 | 123 | 316 | = 2888 |
| **Residue in contact with 2a** | **presence of the contact** | | | | | | | | | **relative occurrence of the contact** [%]^&^ |
| Gly137 |  |  |  |  |  | ***** |  |  |  | 10.6 |
| Tyr139 | ***** |  |  |  | ***** |  |  |  |  | 23.2 |
| **Thr144** |  | ***** | ***** |  |  |  | ***** | ***** | ***** | 51.7 |
| Asn145 |  |  |  |  | ***** |  |  | ***** |  | 16.0 |
| Leu498 |  |  |  |  |  | ***** |  |  |  | 10.6 |
| **Arg499** | ***** | ***** | ***** | ***** | ***** | ***** | ***** | ***** | ***** | 100.0 |
| **Ser500** | ***** |  | ***** | ***** | ***** |  |  |  |  | 50.0 |
| Glu501 | ***** |  |  |  | ***** |  |  |  |  | 23.2 |
| **Pro503** |  |  |  | ***** | ***** |  | ***** |  | ***** | 49.9 |
| **Gln506** | ***** | ***** | ***** | ***** | ***** |  | ***** |  | ***** | 85.2 |
| **Gln538** | ***** | ***** | ***** | ***** |  | ***** | ***** | ***** |  | 77.3 |
| Leu539 |  | ***** |  |  |  | ***** |  |  | ***** | 33.0 |
| Phe541 |  |  |  |  |  |  |  | ***** |  | 4.3 |
| **Arg542** | ***** | ***** | ***** | ***** | ***** | ***** |  | ***** | ***** | 87.3 |
| Glu545 |  |  |  |  |  |  |  | ***** |  | 4.3 |
| Asn546 |  |  |  | ***** |  | ***** |  |  |  | 25.1 |

^&^ Relative occurrence of the contact: total number of structures in the clusters where the residue is in contact with **2a** divided for the total number of structures in the clusters.


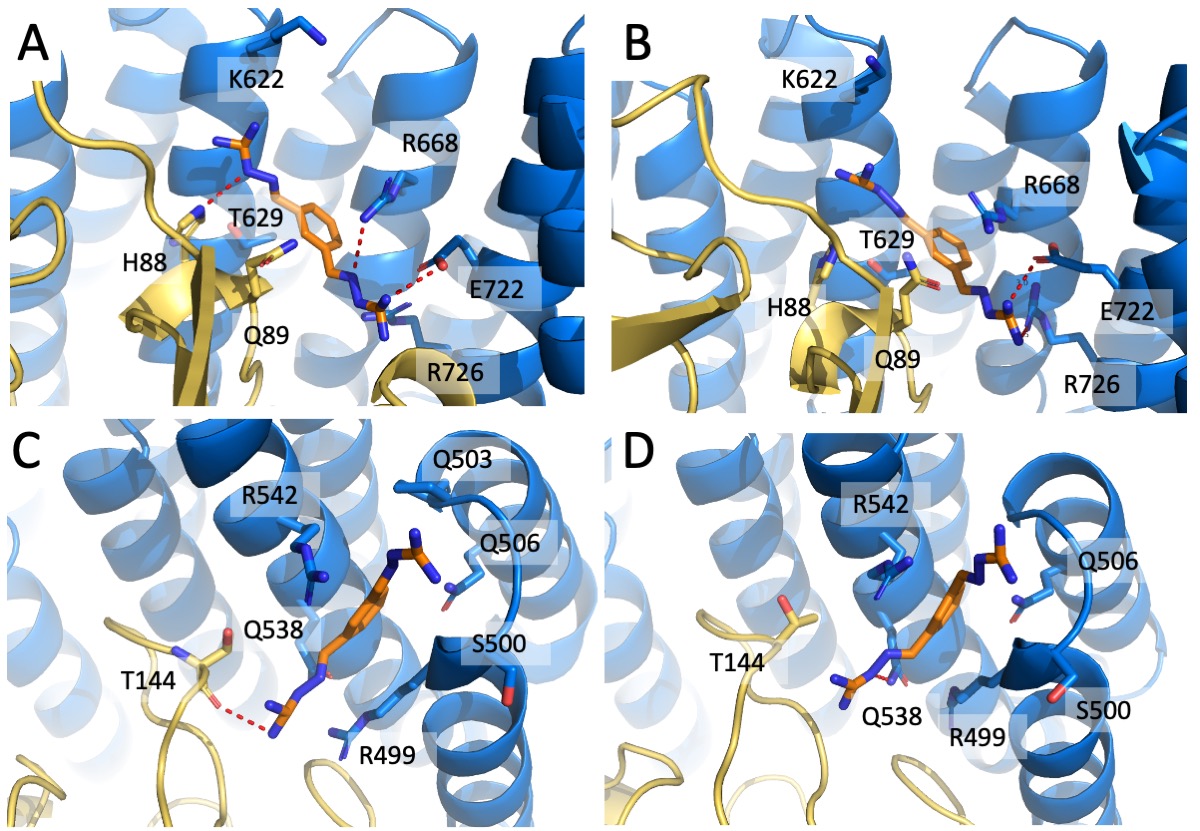


**Figure S8. Representative MD conformations for 2a in *site1* and *site3.*** A and B conformations of the structures of cluster 59.2 and cluster 83.6 for *site1*, respectively*.* C and D conformations of structures of cluster *24.2* and cluster *96.2* for *site3,* respectively.
